# A Novel Highly Divergent Protein Family Identified from a Viviparous Insect by RNA-seq Analysis: A Potential Target for Tsetse Fly-Specific Abortifacients

**DOI:** 10.1371/journal.pgen.1003874

**Published:** 2014-04-24

**Authors:** Joshua B. Benoit, Geoffrey M. Attardo, Veronika Michalkova, Tyler B. Krause, Jana Bohova, Qirui Zhang, Aaron A. Baumann, Paul O. Mireji, Peter Takáč, David L. Denlinger, Jose M. Ribeiro, Serap Aksoy

**Affiliations:** 1 Division of Epidemiology of Microbial Diseases, Yale School of Public Health, New Haven, Connecticut, United States of America; 2 Section of Molecular and Applied Zoology, Institute of Zoology, Slovak Academy of Sciences, Bratislava, Slovakia; 3 Departments of Entomology and Evolution, Ecology and Organismal Biology, The Ohio State University, Columbus, Ohio, United States of America; 4 Janelia Farm Research Campus, Howard Hughes Medical Institute, Ashburn, Virginia, United States of America; 5 Department of Biochemistry and Molecular Biology, Egerton University, Njoro, Kenya; 6 Laboratory of Malaria and Vector Research, National Institute of Allergy and Infectious Diseases, Bethesda, Maryland, United States of America; University of Toronto, Canada

## Abstract

In tsetse flies, nutrients for intrauterine larval development are synthesized by the modified accessory gland (milk gland) and provided in mother's milk during lactation. Interference with at least two milk proteins has been shown to extend larval development and reduce fecundity. The goal of this study was to perform a comprehensive characterization of tsetse milk proteins using lactation-specific transcriptome/milk proteome analyses and to define functional role(s) for the milk proteins during lactation. Differential analysis of RNA-seq data from lactating and dry (non-lactating) females revealed enrichment of transcripts coding for protein synthesis machinery, lipid metabolism and secretory proteins during lactation. Among the genes induced during lactation were those encoding the previously identified milk proteins (*milk gland proteins 1–3*, *transferrin* and *acid sphingomyelinase 1*) and seven new genes (*mgp4–10*). The genes encoding *mgp2–10* are organized on a 40 kb syntenic block in the tsetse genome, have similar exon-intron arrangements, and share regions of amino acid sequence similarity. Expression of *mgp2–10* is female-specific and high during milk secretion. While knockdown of a single *mgp* failed to reduce fecundity, simultaneous knockdown of multiple variants reduced milk protein levels and lowered fecundity. The genomic localization, gene structure similarities, and functional redundancy of MGP2–10 suggest that they constitute a novel highly divergent protein family. Our data indicates that MGP2–10 function both as the primary amino acid resource for the developing larva and in the maintenance of milk homeostasis, similar to the function of the mammalian casein family of milk proteins. This study underscores the dynamic nature of the lactation cycle and identifies a novel family of lactation-specific proteins, unique to *Glossina* sp., that are essential to larval development. The specificity of MGP2–10 to tsetse and their critical role during lactation suggests that these proteins may be an excellent target for tsetse-specific population control approaches.

## Author Summary

Tsetse flies are the sole vector for African trypanosomes, causative agents of sleeping sickness in humans and nagana in cattle. Transcriptome and proteome analyses were utilized to examine the underlying mechanisms of tsetse lactation that occur during each reproductive cycle. These analyses revealed a dramatic shift to the synthesis of milk proteins during lactation and a novel milk-specific protein family. All members of this family were co-localized, shared sequence similarity and were expressed at 40× basal levels during milk secretion. Suppression of gene from this lactation-associated family impaired progeny development by reducing milk protein content and altering milk homeostasis. These novel genes represent an excellent target for tsetse-specific reproductive-based control mechanisms. In addition, the characterization of tsetse milk production revealed multiple factors that are functionally analogous between tsetse and mammalian lactation.

## Introduction

Tsetse reproductive biology is unusual among insects. Female tsetse give birth to a fully mature third instar larva (viviparity) after an extended intrauterine gestation. This reproductive strategy limits the capacity of tsetse mothers to only 8–10 offspring per lifetime [1]. To accommodate intrauterine larval development, the morphology and physiology of the female tsetse reproductive organs have undergone extensive modification. The reproductive tract has been expanded into a uterus to serve as a safe harbor for developing larvae. Ovarian development alternates between the right and left ovaries to produce a single oocyte during each gonotrophic cycle. The female accessory gland has been modified and expanded to provide milk that is secreted into the uterus and consumed by the developing larva [1]. The distinctive aspects of tsetse viviparity represent significant reproductive bottlenecks that could be exploited for population control. Furthermore, identification of factors specific to milk production could lead to development of novel tsetse-specific compounds that interfere with larval development and induce abortion (abortifacients) without impacting non-target insects.

The nutritional components of tsetse milk consist mainly of proteins and lipids emulsified in an aqueous base [2]. In total, 6–10 mg of nutrients (combined with 10 mg of water) are transferred to the larva in the milk during intrauterine development. Few studies have examined regulation of tsetse milk production, including an investigation of structural changes in the milk gland, radioisotope studies of nutrient movement within the mother during lactation, and direct examination of specific milk proteins [1], [3]–[9]. To date, six milk proteins have been characterized, including Transferrin [7], [10], a lipocalin (Milk Gland Protein1, MGP1 [6], [11]), two unknown milk proteins (MGP2–3; [12]), Acid Sphingomyelinase 1 (aSMase1; [9]) and Peptidoglycan Recognition Protein-LB (PGRP-LB, [13]). Furthermore, we recently showed that lipid metabolism is governed by the cooperative activity of insulin and juvenile hormone signaling pathways during the pregnancy cycle [14]. However, the full suite of proteins present in the milk and underlying mechanisms for their regulation during tsetse lactation and pregnancy have yet to be determined.

In this study, a satellite paper to our report on the whole genome sequence of the tsetse species *Glossina morsitans morsitans* [15], we used differential RNA-seq analyses to compare transcript abundance in females carrying an intrauterine larva (lactating) with females 24–48 hours after parturition (non-lactating or dry). The lactation period occurs during larvigenesis, while the dry period occurs over the course of oogenesis and embryogenesis [14]. In addition to transcriptome analysis, protein constituents of tsetse milk were identified through LC/MS/MS analyses of gut contents from nursing larvae. We describe the expression profile, the predicted structure based on an *in silico* approach, and the microsyntenic genomic organization of nine tsetse-specific milk proteins (MGP2–10) that we propose represent a highly divergent, novel protein family. siRNA-based knockdown analysis was employed to examine the functional roles of the MGP2–10 proteins during tsetse reproduction. Since MGP2–10 are tsetse-specific and have substantial influence over tsetse fecundity, we discuss their potential for exploitation in novel population reduction approaches. Lastly, we discuss our findings in light of milk secretions described from other lactating organisms.

## Results

### Identification of genes associated with lactation by transcriptome analyses

To understand the major products of lactation and factors that may be responsible for regulating their expression, we analyzed two RNA-seq libraries. The first library represents lactating females carrying an early third instar intrauterine larva, while the second represents dry females collected approximately 48 hours post-parturition, at which time they had an early embryo developing in the uterus and lactation has yet to commence. In total, over 42 million reads from each of the two libraries were recovered (Table 1). Overall read quality was high for both sample sets based on FastQC analysis. Removal of contaminating tsetse symbiont (*Wigglesworthia*, *Sodalis* and *Wolbachia*) specific sequences and cleanup resulted in a 4% reduction in the total number of sequences identified in lactating flies and a 5.1% reduction for dry flies, respectively (Table 1).

**Table 1:** Total read number generated by Illumina RNA-seq and following quality control measures including symbiont removal and elimination of low quality reads.

	Total reads	Symbiont removal	Trimmed
Lactating	42,036,089	41,436,568 (98.6%)	40,345,695 (96.0%)
Dry	42,206,191	42,122,249 (99.8%)	40,065,395 (94.9%)

**Figure 1:**
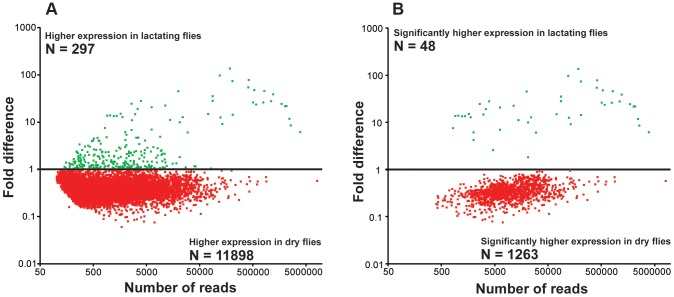
Fold changes in transcript expression for contigs based on RNA-seq analysis. Green indicates expression higher in lactating flies and red indicates higher expression in dry flies. (A) Relative expression of each contig with at least 50 mapped reads. (B) Contigs with significantly different expression values from A (Kal's test with Bonferroni correction, P<0.05).

**Figure 2:**
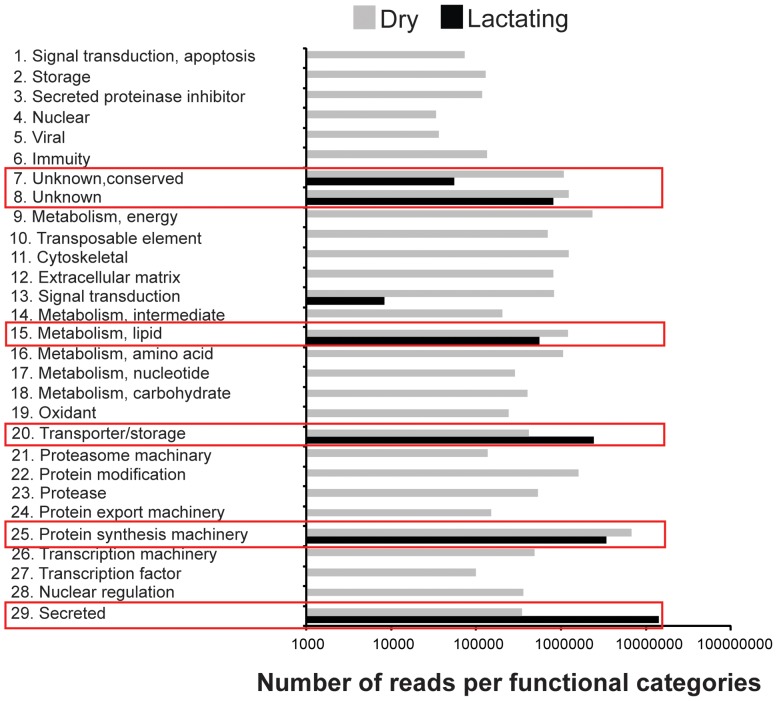
Gene ontology enrichment analysis. Reads in dry and lactating flies that mapped to genes with specific metabolic function.

*De novo* assembly of the two datasets by Abyss [16], [17] and Trinity [18] generated 42,935 contigs that were subsequently identified according to multiple search parameters (Table S2). There were 34,674 contigs at least 200 bp in length, with the longest contig measuring 24,573 bp in size (Fig. S1). Distribution of reads per contig was comparable between the two datasets with the exception that there was a greater number of highly expressed genes in lactating flies (Fig. S2). Comparative analyses of contigs revealed that most were more highly expressed in dry flies compared to their lactating counterparts. A total of 297 contigs (2.1%) with at least 50 mapped transcriptome reads showed elevated expression in lactating *versus* dry flies (Fig. 1a; Table S2). Only 1311 were expressed at statistically different levels between the two datasets, with 48 contigs (4.2%) more highly expressed in lactating flies (Fig. 1b; Table S3). Classification of the lactation-expressed contigs based on specific metabolic and structural functions revealed enrichment for lipid metabolism, transport and storage, protein synthesis, secreted proteins, and those of unknown function (Fig. 2a).

**Figure 3:**
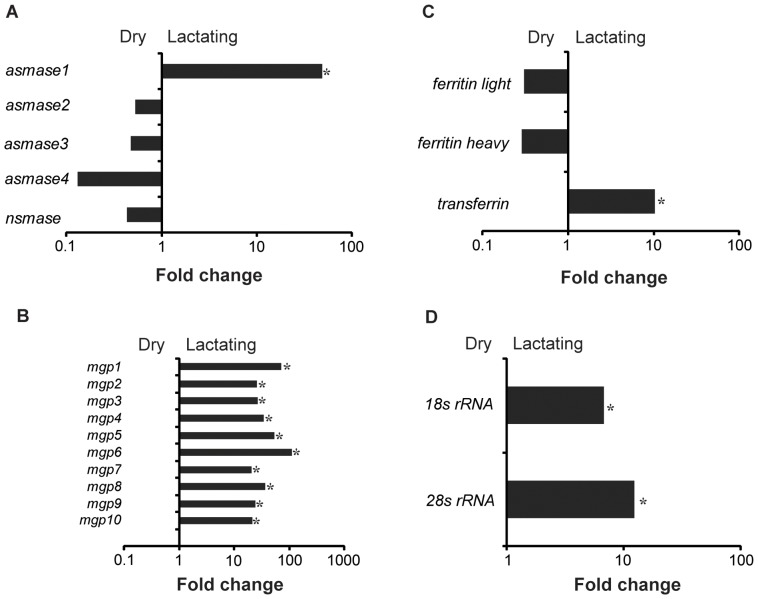
Summary of specific genes that are differentially expressed in lactating compared to dry flies. (A) Sphingomyelinase genes, *asmase1–4* and *nsmase*. (B) Milk gland proteins genes, *mgp1–10*. (C) Iron-associated genes, *non-hemecontaining ferritin*, *ferritin light*, *ferritin heavy* and *transferrin*. (D) Ribosomal RNAs, *18S rRNA* and *28S rRNA*. *, significantly different expression values from B, Kal's test with Bonferroni correction, P<0.05.

Examination of the most highly expressed contigs in lactating flies revealed known milk protein genes along with novel transcripts not previously associated with lactation. The known milk protein genes (*mgp1*, *mgp2–3*, *tsf* and *asmase1*) were expressed at least 10-fold higher in lactating *versus* dry flies (Fig. 3a–c; Table S2, S3). Of particular interest was the discovery of a group of seven new genes similar to the previously identified *mgp2–3* genes that were upregulated during pregnancy (*mgp4–10*; Fig. 3b; Table S2). Transferrin and aSMase1, involved in iron transport and sphingolipid metabolism respectively, were the only other proteins that were highly expressed and showed increased transcript abundance in lactating flies (Fig. 3c; Table S2). Recently, the immunoregulatory Peptidoglycan Recognition Protein LB (PGRP-LB) was also detected in tsetse milk [13]. Based on this analysis, PGRP-LB expression patterns are different from those of other lactation associated-proteins, as its expression did not increase throughout pregnancy (Table S2). In addition to the genes described above, specific ribosomal RNAs were significantly elevated in lactating flies (Fig. 3d; Fig. S3), and may account for the overall increase in contigs coding for genes involved in protein synthesis (Table S2). Confirmation of transcript abundance during lactation was achieved by qPCR analysis of the *mgp1–10*, *28S*, *transferrin*, *pgrp-lb* and *asmase1* genes (Table 2; Text S1).

**Table 2:** Specific proteins documented in tsetse milk based by LC/MS/MS or expressed highly in the transcriptome study.

	Potential milk protein	Fold transcript increase during lactation by qPCR	Function of protein	LC/MS/MS analysis of milk (Empai)
**1**	MGP1	71.93±2.74	lipocalin	3.31
**2**	MGP2	25.52±5.67	unknown	3.45
**3**	MGP3	26.34±6.17	unknown	1.26
**4**	MGP4	34.48±5.85	unknown	1.64
**5**	MGP5	53.58±5.18	unknown	0.28
**6**	MGP6	110.16±16.16	unknown	1.54
**7**	MGP7	20.76±6.27	unknown	8.24
**8**	MGP8	36.55±7.86	unknown	3.38
**9**	MGP9	24.43±2.19	unknown	3.89
**10**	MGP10	21.36±4.34	unknown	2.42
**11**	aSMase1	48.14±12.32	sphingomyelin metabolism	2.33
**12**	Transferrin	10.35±7.86	iron transport	2.01
**13**	PGRP-LB	0.74±0.83	immunity	0.21
**14**	Cu/Zn SOD	0.46±0.16	antioxidant enzyme	0.12
**15**	Catalase	0.53±0.14	antioxidant enzyme	0.09
**16**	Niemann-Pick C- 2g	0.62±0.23	sterol transport	2.01
**17**	Ubash3a-like	0.87±0.34	Immune suppression	3.45
**18**	Gmfb8	0.48±0.29	Unknown	1.26

qPCR validation was performed with a CFX PCR detection system (Bio-Rad, Hercules) and data were analyzed with CFX manager software version 3.1 (Bio-Rad). Data represents the mean ± SE of three replicates and was normalized to *tubulin*.

The majority of the contigs (94%) were more abundant in dry (non-lactating) compared to lactating flies (Fig. 1a; Table S2). Multiple gene families were highly expressed in dry flies (Fig. 2a, Table S2). Contigs encoding heat shock proteins and antioxidant enzymes were increased in dry flies, indicating that dry flies may be better suited than their lactating counterparts to respond to stress and environmental insult (Fig. S4). In particular, qPCR analysis validated the transcriptome data for *Cu/* *Z* *n superoxide dismutase* and *catalase*, which encode proteins that remove reactive oxygen species to prevent damage (Table 2). Lipid metabolism contigs were more abundant in dry flies with the exception of *Brummer lipase*, which was only two-fold higher than in lactating flies (Fig. S4; Table S2). Expression of tsetse yolk proteins, *yolk protein 1–3* (*yp1–3*) was also higher in dry flies, reflecting the yolk protein synthesis that occurs between bouts of lactation (Fig. S4; Table S2). Contigs identified as trypsin showed decreased transcript abundance in lactating flies (Table S4). Given that the transcriptome analysis was from whole females, this finding likely correlates with lactating females' smaller bloodmeals that result from limitations on abdominal space imposed by developing intrauterine larva [5]. These results suggest that many processes in tsetse mothers are down regulated during lactation ( = higher in dry flies), when the female devotes energy and resources to synthesize milk-associated proteins to nourish the developing larva.

**Figure 4:**
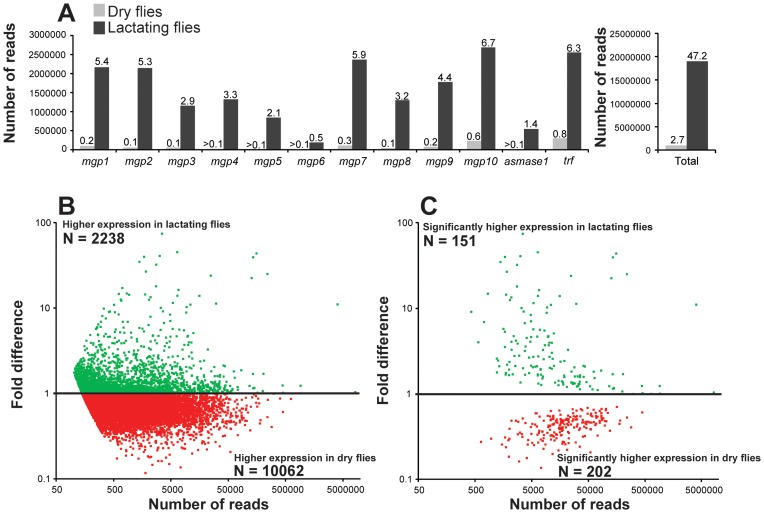
Mapping of reads to lactation-specific genes and fold changes in transcript expression for contigs after removal of lactation-specific genes based on RNA-seq analysis. (A) Left, number of reads mapping to individual genes coding for the 12 milk-specific proteins. Numbers above columns are the percent of total sample that mapped to the specific gene. Right, Sum of reads from all 12 milk-specific proteins. Number above columns are percent of total samples. (B) Relative expression of each contig with at least 50 mapped reads after removal of Illuminia reads for milk-specific contigs. (C) Contigs with significantly different expression values from B, Kal's test with Bonferroni correction, P<0.05.

**Figure 5:**
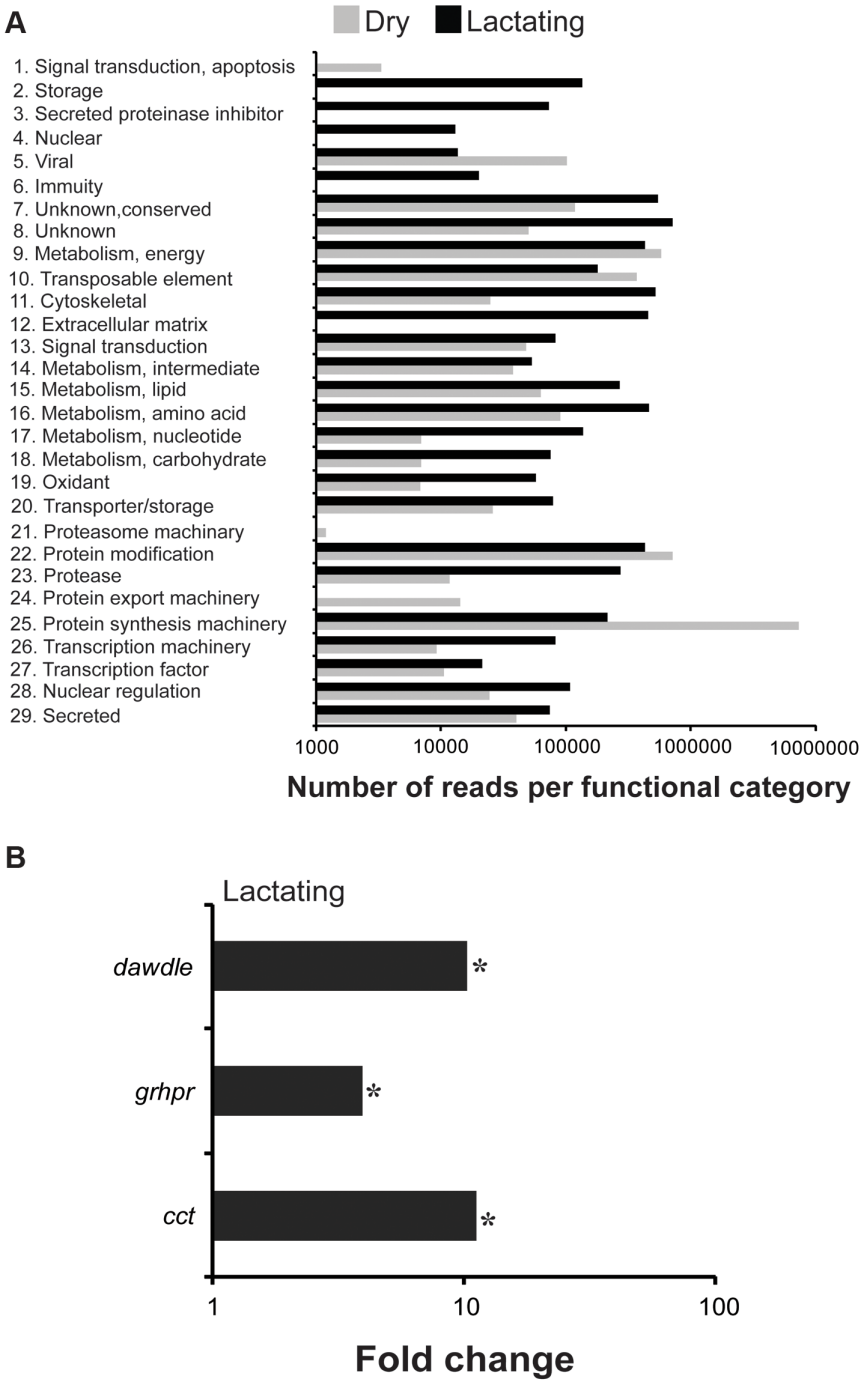
GO enrichment and genes identified following RNA-seq analysis after milk-specific gene removal. (A) Reads in dry and lactating flies that mapped to genes with specific metabolic function. *, indicating a significantly higher level in lactating flies based on chi-square test. (B) Select genes identified as increased during lactation following RNA-seq analysis after milk-specific gene removal. *, indicates significantly different between lactating and dry flies based on Kal's test followed by Bonferroni correction.

We conducted a secondary RNA-seq analysis after removing reads that mapped directly to the twelve most abundant lactation-specific genes (*asmase1*, *mgp1–10* and *transferrin*). Removing these reads yielded only a 2.6% reduction in the dry fly dataset, but a drastic reduction of 47.2% was observed in the lactating fly dataset (Fig. 4a). This difference suggests that lactating flies invest over 47% of their total transcriptional activity toward producing the main protein constituents of tsetse milk (Fig. 4a). Each milk-specific gene accounted for 1.4–6.7% of the total read count in lactating flies, with the most reads mapping to *mgp10* and *transferrin* (Fig. 4a). This removal resulted in a total of 2238 genes that were more highly expressed in lactating flies, but with only 151 that were significantly elevated relative to dry flies (Fig. 4b,c). Assignment by metabolic category resulted in a more balanced distribution of highly expressed contigs in lactating and dry flies (Fig. 5a). This second analysis revealed a few additional contigs whose expression increased during lactation; their expression was previously overshadowed by highly expressed milk-specific genes (Table S5; Table S6). These included *dawdle* (an activin signaling molecule), *glyoxylate/hydroxypyruvate reductase* (an enzyme that converts glycerate to hydroxypyruvate), *choline-phosphate cytidylyltransferase* (an enzyme in the Kennedy pathway that catalyzes choline phosphate to CDP-choline) and multiple ribosomal proteins (Fig. 5b, Tables S5, Table S6).

### Proteins present in tsetse milk as determined by proteome analysis

**Figure 6:**
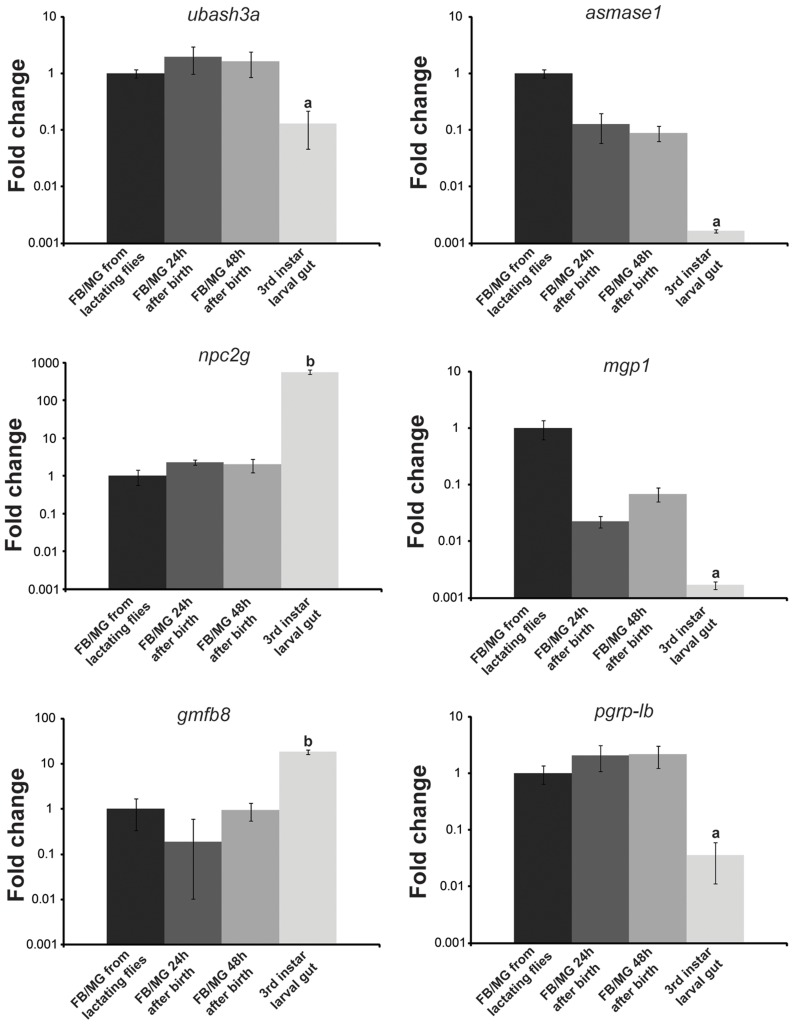
Validation of specific highly abundant proteins within the milk proteome. FB/MG, fat body/milk gland analyzed from lactating flies, 24 hours after birth and 48 h after birth along with 3rd instar larval gut. Transcript levels were determined by qPCR using a CFX PCR detection system (Bio-Rad, Hercules) and data were analyzed with CFX manager software version 3.1 (Bio-Rad). Data represents the mean ± SE of three replicates and was normalized to *tubulin*. a (lower) and b (higher), denotes significant difference by ANOVA with Tukey's test at P<0.01 in comparison to the other samples.

Using LC/MS/MS analyses on the gut contents of feeding larva, we identified 155 proteins that may be constituents of tsetse milk. Most of these proteins have a low exponentially modified protein abundance index (empai) value and are likely present in milk at low levels or may be products from the larval gut (Table S7). Most of the highly abundant proteins identified in tsetse milk were products of genes identified as highly expressed during lactation in our transcriptomics analysis, including MGP1–10, Transferrin and aSMase1 (Table 2). Previously, PGRP was documented in tsetse milk [13] and we confirmed the presence of this immune protein in the milk proteome (Table 2). In addition to the transcriptionally-abundant proteins, the milk proteome identified three other abundant proteins (empai >1.2; Table 2). These three proteins include a sterol binding protein (Niemann-Pick C-2g, NPC2G), Ubiquitin Associated and SH3 Domain Containing A (UBASH3A, a protein belonging to the T-cell ubiquitin ligand, TULA, family [19]), and a putative tsetse protein with unknown function (GmfB8). Transcript levels for NPC2G, GmfB8-like protein and UBASH3A were measured in the milk gland/fat body fraction during and after lactation and in the larval gut to confirm whether these are generated by the milk gland or if they are products of the gut (Fig. 6). Both *npc2g* and *gmfb8-like protein* expression were detected at high levels in the larval gut. Transcript level for UBASH3A was higher in the milk gland/fat body, showing an expression profile similar to PGRP-LB (Fig. 6), suggesting that this is likely a low abundance protein generated by the milk gland during lactation. These results provided further validation for our transcriptome-based identification of milk protein genes as actual secreted products in tsetse milk. In addition, we recovered potential milk proteins that are not under extensive transcriptional regulation during lactation.

### Phylogenetic analysis, gene structure and predicted protein structure of novel milk proteins

**Figure 7:**
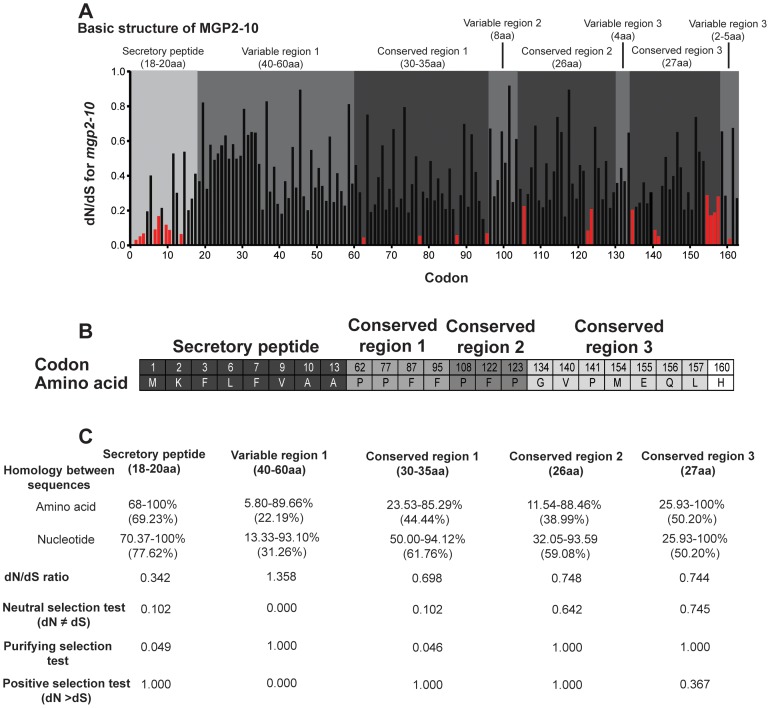
Selective pressure acting upon *mgp* gene family. (A) Site-specific dN/dS analysis along a multiple alignment of MGP coding sequences. Residues identified as subject to negative selection under FEL analysis (posterior probability cutoff = 95) are indicated in red. Regions are described as conserved or variable according to visual examination of the multiple alignment and corroborated by dN/dS anslysis. (B) Specific codons and their corresponding amino acid sequence under negative selection. (C) Percent amino acid and nucleotide homology, average dN/dS ratio and selection tests for specific regions of MGP2–10. Positive, neutral and purifying test were conducted with codon-based Z-test in MEGA5 [25].

**Figure 8:**
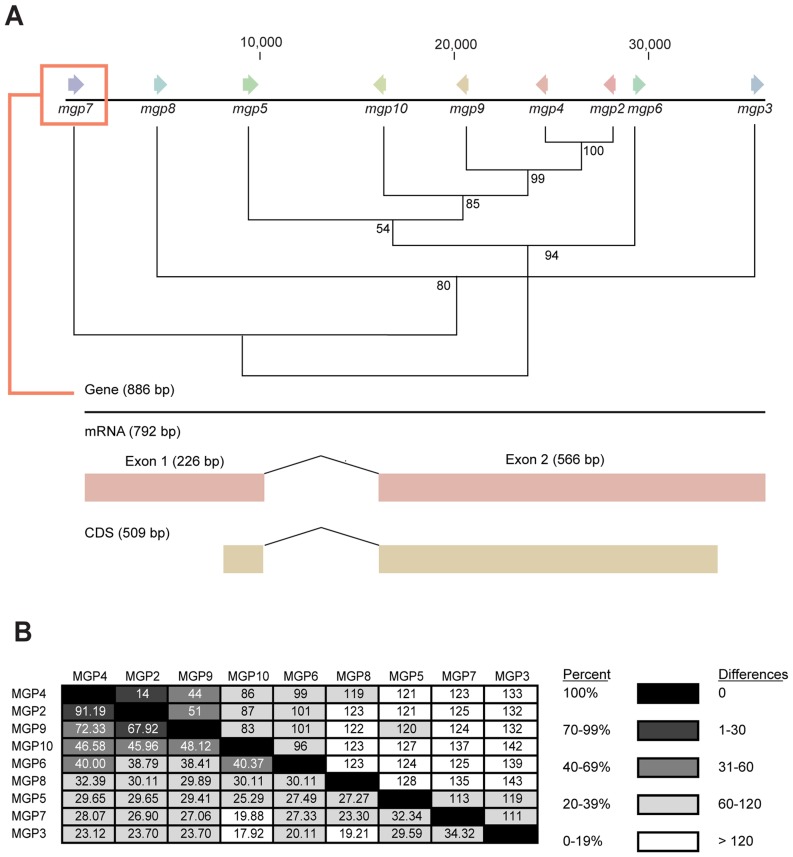
Genome localization of *Glossina morsitans* and *mgp2–10* phylogeny of genes for novel *Glossina morsitans morsitans* milk gland proteins. (A) Initial sequence alignment was completed using ClustalX [23], [124] and formatted with BioEdit [23]. Evolutionary analyses were conducted in MEGA4/5 [25], [26], [125] and displayed as a neighbor-joining tree. (B) Percent amino acid similarity (Bottom) and amino acid differences (Top) between MGP2–10.

BLASTx searches of the NCBI nucleotide collection failed to recover orthologous sequences to the MGP2–10 from any organism. Partial gene sequences encoding MGPs were identified from two other tsetse species, *Glossina fuscipes fuscipes* (MGP2, 5) and *Glossina pallidipes* (MGP3, 4), using RT-PCR with degenerate primers (Fig. S5, S6). Mining of sequence data from recent EST projects on the flesh fly, *Sarcophaga crassipalpis* [20], [21], revealed one sequence with marginal sequence similarity with the tsetse MGP2–10 (Fig. S5, S6). The average number of amino acids for MGP2–10 was 179.3 (range 170–191, Table 3) with a predicted molecular weight of 21.4 kD (range 20.4–22.4 kD; Table 3). The average isoelectric point for MGP2–10 was 6.2 (range 5.9–6.5; Table 3). The aliphatic index, or the relative volume of a protein occupied by aliphatic side chains (alanine, valine, isoleucine, and leucine) that is indicative of the stability of globular proteins [22], is moderate for MGP2–10, ranging from 64–86 (Table 2). There are no predicted glycosylation sites on MGP2–10, but there are at least four predicted phosphorylation sites for each MGP (Table 3). Amino acid alignments of MGP2–10 identified a conserved secretory peptide and three conserved regions with 68–100% and 12–100% nucleotide and amino acid similarity, respectively (Fig. 7a,b; Fig. S5). Phylogenetic analysis shows MGP2 and MGP4 as recently duplicated paralogs sharing 92% amino acid similarity (Fig. 8a; Fig. S6 a,b). Mapping of *mgp2–10* coding sequences to genomic scaffolds revealed that these genes localize to a 40 kb microsyntenic region (Fig. 8a). The phylogeny for *mgp2–10* splits these genes into two distinct groups, one consisting of *mgp2,4,5,6,9,10* and the other of *mgp3,7,8*. When the phylogeny is mapped against the genome location of *mgp2–10*, *mgp2,4,5,6,9,10* localized with a region surrounded by *mgp3,7,8* (Fig. 8a). All *mgp* genes share a conserved exon-intron structure (Fig. 8a), despite showing varying levels of amino acid sequence similarity amongst them (Fig. 8b; Fig. S5). Our results indicate that MGP2–10 proteins are likely specific to *Glossina*, but it remains to be seen if evolutionarily-related sequences may exist in other closely-related viviparous genera (i.e. bat flies and sheep ked; data not available for these species). The sequence obtained from the flesh fly may represent either a class of proteins distinct from the tsetse MGP family, or could be highly divergent ancestral sequence to MGP2–10 genes found in tsetse.

**Table 3:** Characteristics of novel milk gland proteins (MGP2–10).

	MGP2	MGP3	MGP4	MGP5	MGP6	MGP7	MGP8	MGP9	MGP10	Average
**Number of amino acids**	177	187	177	191	176	184	179	170	173	179.33
**Predicted molecular weight (KD)**	21.2	22.06	21.3	22.4	21.46	21.7	21.1	20.4	20.9	21.39
**Predicted glycosylated residues**	0	0	0	0	0	0	0	0	0	0.00
**Predicted phosphorylation sites**	5	5	3	5	6	6	6	4	9	5.44
**Theoretical isolelectric point**	6.06	6.20	6.06	5.86	6.31	6.92	5.96	6.03	6.46	6.21
**Aliphatic index**	64.46	82.78	64.46	87.81	66.59	85.76	76.26	71.11	68.21	74.16

Theoretical isoelectric, predicted molecular weight and aliphatic index were determined with CLC Main Workbench (CLC Bio). Glycosylation sites were predicted by NetNGlyc 1.0 [130] and NetOGlyc 3.1 [131] Predicted phosphorylation sites were identified by NetPhos 2.0 [132].

Based on amino acid composition, the novel tsetse milk proteins provide all essential amino acids necessary for larval growth and development (Table S8). Protein structure predictions for the novel MGPs were generated by four individual programs. Structural predictions were *ab initio* as no homologous protein structures were available. The *ab initio* structure predictions from the four programs revealed that MGPs usually form multiple α-helices (6–10 per protein). However, no functional insights were provided by the I-TASSER program, since MGPs lack structural similarity to other characterized proteins (Fig. S7). Of particular interest, these proteins contain high percentage of hydrophobic amino acids (this study, [12]), including the hydrophobic secretory peptide that was identified in tsetse milk, indicating that this region is not always cleaved during secretion.

Examination of amino acid alignments identified several regions of moderate conservation across MGP2–10 from *G. morsitans*. To assess the relative selective pressures acting on these paralogous genes, we performed several computational analyses of nonsynonymous-to-synonymous substitution ratios (dN/dS) along the coding sequences for these genes. This type of analysis is usually conducted on orthologous genes in different species or on multiple alleles within a species, but utilization of this on paralogous genes could provide insight into regions critical to their function. When dN/dS substantially exceeds 1, evidence for positive selection ( = adaptive evolution) is inferred. In contrast, dN/dS = 1 implies neutral evolution, while dN/dS values closer to zero provide evidence for negative or purifying selection. Sequences were translated and multiple alignments were performed in ClustalX [23], followed by optimization in BioEdit [24] or MEGA 4/5 [25], [26]. We reverse-translated amino acid sequences to obtain codon alignments as input sequences for dN/dS analyses under both PARRIS [27] and FEL (Fixed Effects Likelihood; [28]) analyses in DataMonkey (www.DataMonkey.org; [29], [30]), a web-based implementation of the HyPhy algorithm [31]. PARRIS allows detection of positive selection across an entire coding sequence, while the FEL method is suitable for detecting positive or negative selection in a site-specific manner in small (10–15 sequences) datasets [28]. Under the PARRIS algorithm, we found no evidence for positive selection across the coding sequence of MGP2–10, suggesting that no residues in these proteins are targets of adaptive evolution. FEL analysis likewise showed no evidence for individual codons subject to positive selection. In contrast, while the preponderance of residues in MGP2–10 are apparently undergoing neutral evolution, FEL analysis indicates that the identified N-terminal secretory signal peptide is largely subject to purifying selection (Fig. 7a,b), suggesting that this region is indispensible for protein function or appropriate intra/intercellular transport. A minority of residues, largely dispersed throughout the C-terminal half of MGP2–10 are additionally subject to negative selection as evidenced by dN/dS ratios significantly less than 1 (p = 0.05, Fig. 7a,b). A role for amino acids under purifying selection outside of the secretory region is unknown. A majority of these conserved sites are proline (33.3%) and phenylalanine (26.6%) residues, suggesting these amino acids may be critical for MGP2–10 folding and/or function.

Previous examination of mammalian milk proteins revealed that discrete, specific sections of each gene are subject to neutral, negative or positive selection [32]. Using a similar MEGA-based analysis to specifically investigate MGP2–10 from *G. morsitans*, the secretory peptide and conserved region 1 appear to be largely under negative selection (Fig. 7b). The first variable region has a high dN/dS and is likely subject to neutral or positive selection (Fig. 7b), but additional MGP sequences need to be recovered from other *Glossina* sp. to more confidently determine site- or region-specific selective pressure across MGP coding sequences. Overall, these analyses indicate that only the secretory peptide and the first conserved region are likely subject to purifying selection, but additional analysis will be necessary once full-length MGP genes are recovered from other members of *Glossina* to establish regions of selection.

### Novel milk proteins are specific to female reproduction

**Figure 9:**
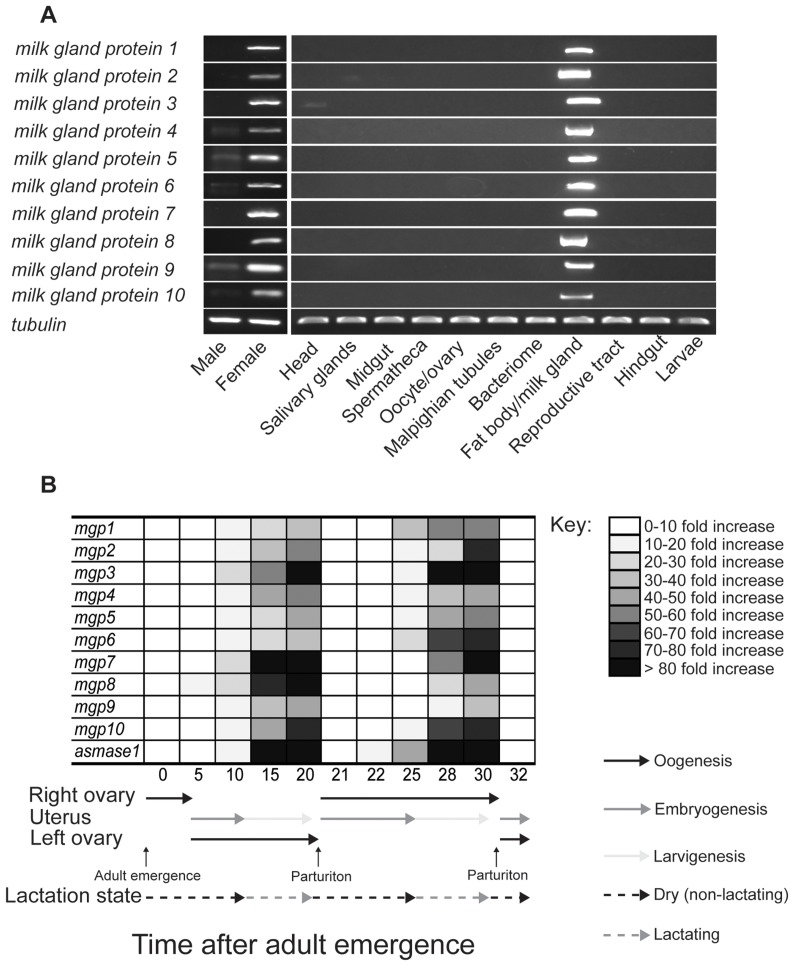
Temporal and spatial expression of milk gland protein genes. (A) Tissue specific RT-PCR. Data represents three replicates. ( B) Time course of *mgp1–10* and *asmase1* expression during the first two tsetse gonotrophic cycles. Transcript levels were determined by qPCR using a CFX PCR detection system (Bio-Rad, Hercules) and data were analyzed with CFX manager software version 3.1 (Bio-Rad). Data represents the mean ± SE of three replicates and was normalized to *tubulin*.

Using RT-PCR analysis to determine the tissue specificity of MGP expression, we found that expression of *mgp2*–*10* is specific to the female fat body/milk gland (Fig. 9a). Temporal expression profiles obtained for these genes showed that *mgp2–10* transcripts increase dramatically during larvigenesis and then rapidly decline within 24–48 h following parturition (Fig. 9b). This temporal and spatial expression profile is consistent with other characterized milk proteins, including *mgp1* (Fig. 9a, [6]), *asmase1* [9] and *transferrin* [7]. The temporal expression profiles for the MGP genes we identified from the two other tsetse species (*gpmgp3,4* from *G. pallidipes* and *gfmgp2,5* from *G. fuscipes*) were similar to those observed for *mgp2–10* in *G. morsitans*. Transcript abundance was lower in teneral females and in females with developing intrauterine embryos, becoming progressively greater through larvigenesis (Fig. S8). In contrast, the MGP-like sequence discovered from the flesh fly, another brachyceran that exhibits larviposition but does not nourish the developing larva, was not expressed in this manner; we observed no differences in MGP-like gene expression between male and female flesh flies (nonpregnant vs. pregnant minus larval expression level; Fig. S8). Thus, even though a gene with moderate sequence similarity was identified in *S. crassipalpis*, its expression profile is incongruent with tsetse MGPs.

### MGP2–10 are critical components of tsetse milk

**Figure 10:**
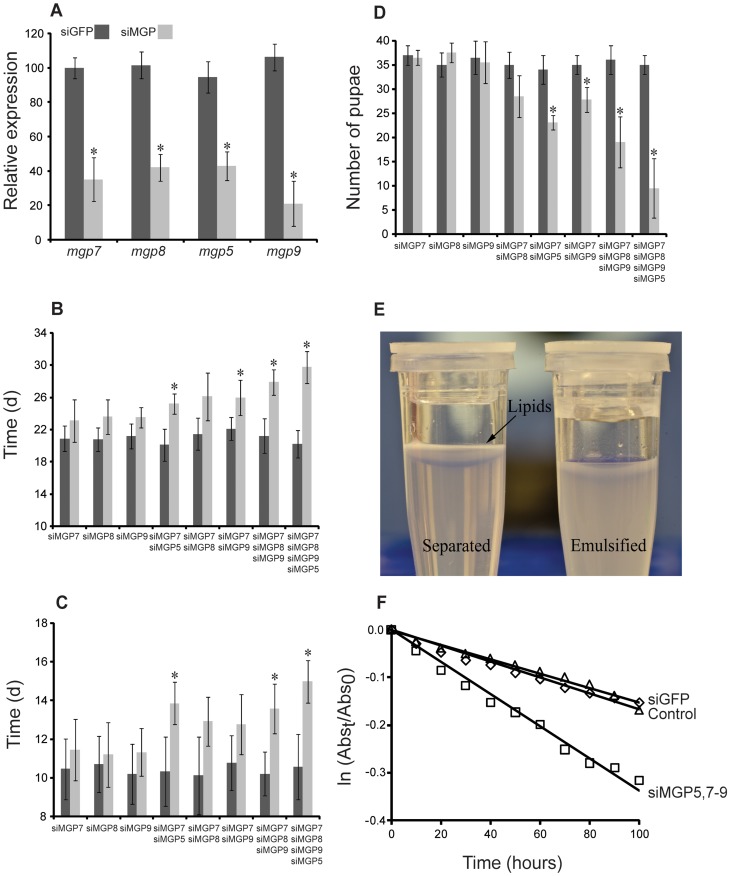
Phenotypes in lactating females following injection of MGP-specific siRNA. (A) Transcript levels determined by qPCR after siRNA injection, mean ± SE of three groups of 3 combined flies normalized to *tubulin*. (B) Duration of the 1st gonotrophic cycle after siRNA injection, mean ± SE of three groups of 30 flies. (C) Duration of the 2nd gonotrophic cycle after siRNA injection, mean ± SE of three groups of 30 flies. (D) Number of pupae deposited by 20 females over 40 d (Only those from the first two gonotrophic cycles were counted), mean ± SE of four groups of 20 flies. (E) Example of lipid separation from an unstable tsetse milk emulsion. (F) Rate of emulsion separation after MGP7–9 knockdown, mean ± SE of ten assays. *, denotes significant difference from siGFP-injected control following ANOVA with Tukey's test at P<0.01.

Injection of siRNAs targeting *mgp5,7–9* significantly reduced corresponding target transcripts in lactating flies (Fig. 10a). Differences in the knockdown efficiency are likely due to the combined effects of technical variation and slight natural variation in the pregnancy cycle. Suppression of individual transcripts (even the highly expressed *mgp7*) had no effect on the number of pupae produced per female, the length of pregnancy, or the incidence of pupal emergence (Fig. 10b–d). This suggests functional redundancy among the multiple *mgp* paralogs, which are all expressed in a similar spatio-temporal manner during pregnancy. Simultaneous suppression of two MGPs (i.e. 5 and 7) reduced the number of pupae deposited per female by 10–15% and extended the duration of pregnancy by 2–3 d, but no difference was observed in the incidence of adult eclosion (Fig. 10b–d). When *mgp5,7–9* were co-suppressed, fecundity was reduced by nearly 70% and in cases where mothers produced viable progeny, pregnancy was extended by 6–8 d (Fig. 10b–d). Together, these results suggest that the paralogous *mgp*s share a critical function in tsetse reproduction.

Bradford assay of the milk protein content indicated that simultaneous knockdown of *mgp5,7–9* reduced overall milk protein by nearly 22% (0.43±0.06 mg protein/5 µl milk) compared to siGFP treated controls (0.56±0.04 mg protein/5 µl milk). We hypothesized that these proteins may serve a function in maintaining milk lipid stability. To explore this possibility, we assessed the stability of milk emulsification after MGP knockdown utilizing an emulsion stabilization assay. Knockdown of *mgp5,7–9* resulted in an increased rate of separation of the aqueous and lipid fractions of the milk by over two-fold (Fig. 10d,e). These findings suggest that the novel MGPs are not only an important amino acid/protein resource for the developing larva, but function to stabilize lipids within tsetse milk, allowing fat to remain homogenously distributed.

## Discussion

Human African Trypanosomiasis (HAT; sleeping sickness) is a fatal disease that affects millions of people in sub-Saharan Africa. A disease caused by closely related parasites in animals, known as African Animal Trypanosomiasis (AAT; nagana), devastates agricultural and livestock production systems as well as land use in Africa. There are no prophylactic drugs that are affordable and highly efficacious, and simple tools for diagnosis or mammalian vaccines for control of HAT or AAT are lacking. Tsetse (*Glossina* sp.) are vectors of African trypanosomes and reduction of this insect population is a highly effective control strategy. The success of population reduction based control efforts largely is due to the low reproductive capacity of tsetse, which is viviparous yields few progeny per female over their lifespan.

Our main goals in this study were to provide an in-depth characterization of the tsetse lactation process, which is essential for intrauterine larval development and to discover novel biochemical, molecular or physiological targets for tsetse population control through reproductive suppression. Through transcriptomic analysis we identified twelve major tsetse milk proteins (MGP1–10, aSMase1 and Transferrin). Expression of these genes is tightly regulated through the transition between dry and lactating periods in order to optimize resource allocation for milk production. We also analyzed milk collected from larval guts using a proteomics approach both to characterize its composition and to verify secretion and transfer from mother to offspring of the nine milk proteins in addition to Transferrin, aSMASe1 and MGP1. Among the proteins identified as lactation products, MGP2–10 represent a group of secreted proteins unique to tsetse. Using a knockdown approach we showed that tandem suppression of multiple *mgp* variants resulted in substantial delays in parturition and up to a 70% reduction in fecundity. Likely, this impaired fecundity results both from a lack of protein resources and impaired lipid stabilization in the milk emulsion.

### Transcriptome analysis reveals a shift in metabolism to milk production during larvigenesis

Several of our major findings from this transcriptome analysis included evidence for a substantial shift during tsetse milk production toward secreted proteins, genes involved in lipid metabolism, transporter genes, and specific genes coding for protein synthesis machinery. Structural studies have previously shown that there are extensive arrays of endoplasmic reticulum (ER) that develop in actively secreting milk gland cells, and then degenerate during an involution period following parturition [33]. A similar pattern of increased ER production in the milk gland was documented in another tsetse fly species, *Glossina austeni* [34] and in *Melophagus ovinus*, a viviparous sheep ked [35]. The rate of protein synthesis is elevated in the milk gland during periods of lactation, in accordance with an increase in ER [1], [36]–[38]. Increase of milk gland associated ER to allow for production of lactation-associated proteins is likely the reason for high abundance of transcripts for genes involved in protein synthesis during tsetse milk production. The products of *mgp1–10* likely constitutes over 95% of the protein content in tsetse milk (this study, [6], [12]), accounting for the increase in contigs for secreted proteins. Transferrin and aSMase1 account for the high read abundance of contigs associated with transporters and lipid metabolism, respectively.

The extreme elevation of *asmase1*, *mgp1–10* and *transferrin* transcript levels during lactation indicate that the expression of these 12 genes which constitutes less than 0.0005% of the contigs from the *de novo* library, represents over 45% of the RNA-seq library. In contrast, in dry flies, these same gene transcripts represent less than 2.6% of the library. A heavy investment in specific genes during reproduction is not uncommon [39]–[43], but in most studies the effect was measured directly within a specific organ, rather than the entire organism. As an example, the lactating wallaby invests over 50% of transcript abundance in the mammary gland to the production of protein bound for transfer in the milk [39]. In addition, most milk production transcripts, specifically those directly incorporated into milk, show drastic changes throughout lactation in mice and bovine within the mammary gland [44]–[46]. For tsetse, heavy transcript investment according to total Illumina read levels is based on the entire female fly rather than the isolated milk gland organ. This high investment is not surprising since at least 4–5 mg (20–25% of the total mass of the mother) of proteins are secreted by the milk gland during the 4–5 day period of lactation, representing 40–50% of the nutritional content of the milk [2]. Thus, female tsetse may be uniquely adapted to generate milk with its entire resources devoted to the transcription of milk proteins at the expense of other biological processes during lactation.

Increases in *transferrin*, *asmase1* and *mgp1* expression during lactation were expected since these proteins are recognized as major constituents of tsetse milk [7]. The role for Transferrin in tsetse milk has yet to be determined [7], though speculation suggests transferrin may serve as a source of iron as well as for immune development/protection [7]. Regarding other milk proteins, knockdown of *asmase1* in lactating females reduces fecundity and severely impacts progeny fitness [9]. Biochemical studies have revealed that secreted aSMase1 is inactive and conversion to the biologically active form, which allows sphingomyelin digestion, occurs upon encountering the acidic conditions of the larval gut [9]. As a lipocalin, MGP1 likely carries a critical unknown hydrophobic ligand in the milk [6], and has been documented to be critical for tsetse fecundity [6].

Lipids, specifically triacylglycerides, constitute the other major nutritional components in tsetse milk [2], [47]. Recent studies have shown that Brummer (Bmm) lipase- and adipokinetic hormone (AKH)-mediated lipolysis are both critical for mobilizing lipids during tsetse reproduction [47]. Our transcriptome data indicate that only a single lipase, *bmm*, is increased during lactation, while expression of most other lipid metabolism genes are suppressed or expressed at levels equivalent to those seen in dry flies. Such minimal transcript variation is perhaps not surprising in light of recent studies on insect lipolysis, which reveal that most control occurs at the post-translational level, either through insulin signaling or through other factors that interact with the surface of the lipid droplet [48]–[51].

An argument for post-translational regulation is further supported by our recent study showing that insulin and juvenile hormone signaling both act to coordinate lipid metabolism in tsetse mothers through transcriptional regulation of select lipolytic/lipogenic genes including *midway* and *bmm*, while other such genes associated the lipid metabolism show little variation [14]. Further, a reasonable explanation for the lack of an observed increase in lipolysis genes is that these genes typically increase prior to lactation (late embryogenesis/early larvigenesis [14]), while our lactating sample was collected during the peak of lactation occurring during the last stages of larvigenesis. In addition, *perilipin1* and *perilipin2* transcripts are elevated in dry versus lactating flies and perhaps these proteins, which interact with lipid droplets, are necessary to accommodate the drastic increases in fat body volume that occurs during the involution periods that separate lactation cycles. In general, our results regarding expression of lipid metabolism genes support our previous studies that *bmm*-mediated lipolysis plays a critical role in regulating lipid homeostasis during pregnancy [14], [47].

Removal of reads mapping to the 12 abundant milk protein genes during RNA-seq analysis allowed for the identification of three additional genes that were enriched and highly expressed during milk production. Dawdle is an activin signaling molecule that has been linked to synaptic growth at the neuromuscular junction [52] and immunity [53] in *Drosophila*. As a member of transforming growth factor beta (TGF β) superfamily of growth factors, activin may be signaling growth of a specific tissue, possibly the milk gland, during lactation. In addition to the role in *Drosophila*, activin is key in regulating growth of the mammary gland during lactation in multiple mammals and has a critical role in breast cancer [54]–[56]. The increased levels of *glyoxylate/hydroxypyruvate reductase*, *grhpr*, may provide additional substrates to maintain homeostasis of proline, the main nutrient source in tsetse hemolymph [57]–[59], as milk production requires a massive amino acid investment [1]. Finally, expression of choline-phosphate cytidylyltransferase, *cct*, has been linked to changes in lipid droplet size [60], and this enzyme may be playing a role either in the fat body during the rapid lipolysis associated with tsetse lactation [47], or in the generation of fat globules for incorporation into the milk. Alternatively or in combination, CCT could be critical for the allocation of choline and choline-derivatives into milk during lactation. The provision of choline is essential for proper organismal growth and development [61], [62].

Our transcriptome data revealed that the majority of genes are expressed at higher levels in dry *versus* lactating flies. This difference is likely due to the fact that transcript levels for most genes are reduced in lactating flies at the expense of generating lactation-specific proteins. In dry flies, transcript elevation for genes associated with digestive processes likely corresponds to the increased bloodmeal size in flies not harboring an intrauterine larva [1]. Elevated transcripts for genes coding for heat shock proteins suggest that dry flies may be better suited for stress tolerance than their lactating counterparts. In addition, proteins involved in oocyte development are elevated in dry flies, likely since oocyte development is nearly complete before lactation begins [1]. Thus, the transcript profile diversity in dry flies is more robust, featuring a more global/representative expression of genes, compared to the rather specific gene set expressed in lactating flies.

### Proteome validates highly expressed milk genes and identifies potential minor milk constituents

Recent studies focusing on MGP2 and MGP3 failed to verify their transfer to the nursing larvae since antisera were not available [12]. The proteomic analysis performed here confirms that these highly expressed genes synthesize milk proteins that are indeed transferred to the intrauterine larva. The proteomics data also confirm that Transferrin, MGP2–10, and aSMase1 are the primary protein components of the tsetse milk [6], [7], [9]. In addition our proteomic analysis also identified UBASH3A as a component of the tsetse milk. UBASH3A is a member of the TULA protein family and contains ubiquitin-associated (UBA) and Src-homology 3 (SH3) domains along with a histidine phosphatase domain [19], [63], [64]. A potent regulator of cellular function documented in most metazoan species [19], [64], UBASH3A is critical for regulation of T-cell proliferation and other aspects of the mammalian immune response, specifically for suppressing immune cell proliferation. The role of insect UBASH3A has not yet been determined but the presence of UBASH3A in tsetse milk suggests that it may play a role in modulating the immune system of the mother or progeny to allow intrauterine larval development. Along with potentially modulating mother-offspring immune relationship, tsetse's milk secretions also provide a route for the transmission of tsetse's microbial symbionts (*Wigglesworthia* and *Sodalis*, [65], [66]) and host immune responses may need to be regulated for symbiotic homeostasis. Our prior studies had shown that the presence of PGRP-LB in the milk is critical for symbiont transfer and overall offspring fitness [13] and the presence of UBASH3A may play a similar role in host-symbiont dynamics during the bacteria transfer within the milk. The ability to transfer symbionts to allow for maintenance of the microbiome in the offspring has been documented to be critical for tsetse immune maturation [67] and the development of the peritrophic matrix development [68]. Many other proteins were observed at lower levels; these low abundance proteins may be critical for larval development. Due to the recovery of milk from within the larval gut contents, we cannot rule out the possibility that these proteins could be products of the larval alimentary canal. Studies devoted to each low abundance peptide will be necessary to determine if it is a product of tsetse milk.

### Identification of a highly divergent protein family as a critical component in the milk secretion

We identified seven new milk gland proteins, MGP4–10, that are similar to MGP2–3. MGP2–10 each contains a conserved secretory signal and multiple sites throughout three moderately-to-highly conserved regions with several residues under apparent strong purifying selection. Structural analysis of these MGPs failed to provide functional insights, but did reveal that these proteins are likely globular, consisting of multiple α-helices. Further study is necessary to conclusively determine the structures of these novel proteins. The coordinated high expression levels observed for *mgp2–10* during lactation and reduced expression after parturition indicate that these proteins are under similar transcriptional regulation and that they may also serve as a source of proteins for larval nutrition [this study, 12]. Indeed, milk protein content was reduced by 20–25% when *mgp5,7–9* transcripts were suppressed by 60–70%, suggesting that MGPs, based on total transcript abundance, likely account for 70–75% of the total protein content of tsetse milk. The MGP2–10 proteins also contain all amino acids, supporting the notion that they function as a complete protein resource for the developing larva. Furthermore, multiple phosphorylation sites associated with each protein suggests that MGP2–10 may also serve as a source of phosphate in tsetse milk. The lack of predicted glycosylation sites on MGP2–10 is not surprising since carbohydrate levels are extremely low in tsetse milk [2].

Previous studies have shown that low molecular weight proteins interact with lipids in tsetse milk [2]. This prompted us to investigate a potential role of MGPs for stabilization of milk-borne lipids. Here, we show that RNA interference of *mgp7–9* results in acceleration of lipid separation from the aqueous phase of milk. This suggests that MGP7–9 (and likely the other MGPs) may represent the previously-documented, unidentified low molecular weight proteins associated with tsetse milk lipids [2]. MGP2–10 have a high proportion of hydrophobic amino acids [this study, 12], which may enable these proteins to interact with milk lipids. Thus, it appears that these newly-identified proteins are critical for maintenance of proper lipid/water dynamics in tsetse milk.

Similarities among MGP2–10 suggest that these proteins represent a highly divergent lactation-specific protein family from tsetse flies. These genes are localized to a single 40 kb chromosomal loci, have similar gene structures and their phylogeny correlates with their chromosomal organization indicating that *mgp2–10* may have expanded by multiple gene duplication events from a common ancestor. It is possible that ancestral duplication events yielded two separate groups which may have been subsequently expanded as a result of unequal genetic crossing-over with the *mgp2,4,5,6,9,10* being encoded on the antisense strand. Predicted three-dimensional structures between MGP2–10 is similar including multiple α-helices and a globular protein tertiary arrangement. *mgp2–10* are under nearly identical transcriptional regulation showing increased expression during tsetse fly lactation and rapid decline during involution. These proteins also exhibit functional redundancy as a source of secreted amino acids in the milk and in sustaining lipid-protein homeostasis within the aqueous milk base. Although MGP2–10 have varying levels of amino acid similarities (18–91%), there are conserved regions they share outside of the secretory peptide. Specifically, 23 sites are under purifying selection (8 in the secretory peptide coding sequence and 15 dispersed throughout the remaining portions of the sequence), and these are likely critical to the functional role of MGP2–10 during tsetse lactation. Collectively, similarities between MGP2–10 indicate that these proteins constitute a novel family in tsetse similar to other highly divergent protein families, including caseins [69], [70], aquaporins/major intrinsic proteins [71], [72], odorant binding proteins [73], [74] and small heat shock proteins [75].

### Comparative biology: Tsetse *vs.* mammalian lactation

Our previous work demonstrated that several mechanisms underlying tsetse lactation parallel characteristics of mammalian lactation. First, both systems have highly specialized lactating cells that cycle through periods of high productivity during lactation to low activity following involution [76], [77]. Second, there are multiple, functionally analogous proteins involved in tsetse and mammalian lactation [78], [79]. These proteins include a lipocalin (MGP1 vs. β-lactoglobulin [6], [38], [45], [80]), an iron-transfer protein (Transferrin vs. Lactoferrin [7], [10]), SMase in milk or the gut contents of feeding progeny [9], [81]–[83] and various immunity proteins (PGRP and UBASH3A vs. multiple mammalian immunity proteins, [this study], [ 45], [78], [84,85]. Third, the lipid content transferred to the developing offspring is similar during lactation in both systems. Fourth and finally, microbial symbionts are transferred from the mother to the developing offspring in both tsetse and mammals [66], [86]–[88]. There are however a few noteworthy differences between tsetse and mammalian lactation, such as the abundance of calcium transport proteins in mammalian not found in tsetse milk [this study], [76], [79,89]. This difference is unsurprising, since insects do not require large amounts of calcium for their chitin-based exoskeleton. In addition, tsetse milk contains a lower carbohydrate content than mammalian milk [76], [90], indicating that tsetse flies rely solely on lipids and protein for growth and development, rather than a combination of sugar/lipids/protein as in the mammalian case. Such reduced reliance on sugar is also unsurprising as tsetse flies have little to no detectable levels of glucose within their bodies and use proline as their circulating hemolymph resource, rather than a glucose-based substrate such as trehalose [1], [57].

Mammalian genomes contain no orthologous sequences to the nine novel tsetse MGPs. However, MGPs might function analogously to caseins in mammalian milk. Caseins are the major amino acid and calcium source for the mammalian neonate [65], [70], [91]. While MGPs do not carry calcium, they do, like caseins, represent a major amino acid resource in the milk [39], [46], [69], [92]. The presence of multiple phosphorlyation sites in MGPs suggests that this novel protein family may also act in tsetse milk as a source of phosphate as do caseins in mammalian milk [69], [70]. Furthermore, caseins are amphipathic molecules that form micelles, which interact directly with lipids both *in vivo* and *in vitro* [69], [93]. According to our results, MGPs likewise interact with lipids to promote stability of lipid emulsions in the aqueous tsetse milk. To determine if MGP2–10 have amphipathic structural properties like caseins, direct protein structural studies, rather than protein modeling, will be necessary. In addition, expansion of the casein and MGP gene families has occurred for both mammals and tsetse within specialized regions of their genomes. This indicates that expansion of these protein families (MGPs and caseins) is advantageous for provisioning the necessary nutrients in both tsetse and mammalian milk, respectively [this study], [ 69,92]. Finally, members of the MGP and casein families show substantial divergence in sequence similarity [this study], [69,78], which is a characteristic of proteins that are mainly nutritional components of milk. Proteins involved in mechanics of lactation, i.e. milk fat globule formation or have an enzymatic function, are typically more conserved within and between organisms [78]. These similarities further support the idea that MGPs perform an analogous role to mammalian caseins in tsetse milk.

Few studies have examined the effects of casein knockdown/knockout in mammals. In mice, knockout lines have been developed for α-, β- and κ-casein [94]–[96], and in goats there are naturally occurring deficiencies in α-casein [97]. Knockdown phenotypes differ dramatically, depending on the casein variant targeted. The knockout mutant for β-casein in mice [95] and null α_S1_-casein in goats [97] have no or minimal apparent effects on milk production, potentially due to increased expression of other casein genes to compensate for the loss of β-casein or α_S1_-casein, respectively. Offspring receiving milk from α-casein-null mothers experience delayed growth and life-long body size reduction, but only transient effects on physical and behavioral development [96]. The most drastic change is noted in κ-casein null mice, which fail to lactate [94]. Similar to suppression of caseins, knockdown of individual tsetse MGPs had only minimal effects on tsetse fecundity; more drastic changes occurred upon silencing multiple transcripts. In addition, a reduction in tsetse MGPs accelerated separation of lipid emulsions. Caseins likely interact similarly with lipids in mammalian milk to promote lipid emulsifications. Indeed, in addition to their biological roles, caseins have also been industrialized as emulsifying agents [98], [99]. This feature highlights the ability of these proteins to stabilize lipids present in the milk, as noted in tsetse. Proteomic studies examining mammalian milk fat globules have identified caseins, indicating that these proteins are associated with milk lipids [100]–[102]. Specifically, casein modification alters lipid composition and protein components of the milk fat globule in goats [103]. The analogous functions of MGP2–10 and caseins suggest roles for these proteins as a source of amino acids, as stabilizers of milk homogeneity, and as carriers of polyatomic ions (i.e. phosphate groups). These roles must be fulfilled by a specific abundant protein or protein family to satisfy nutritional requirements of an immature organism during periods of lactation.

### Conclusions

**Figure 11:**
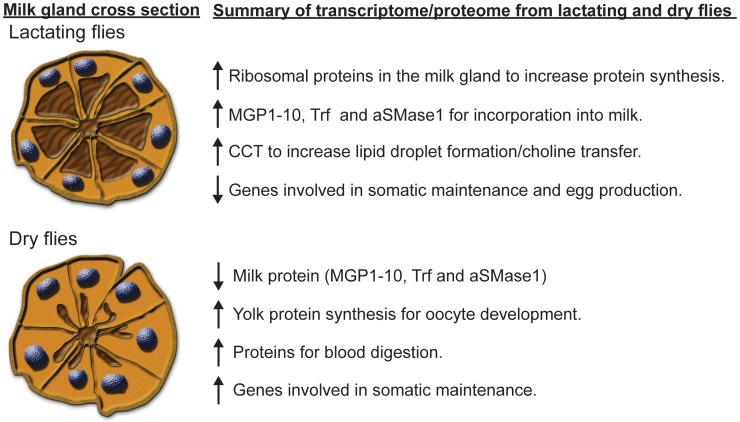
Summary of the results from our tsetse fly lactation study. The cross section of the milk gland tubules was adapted from Yang et al. [12] and modified according to Ma et al. [33] to represent tubules in a lactating fly, characterized by secretory vacuoles full of milk and condensed nuclei, and in the milk gland of a dry fly, characterized by exhausted secretory vacuoles and expanded nuclei.

This study provides the first complete examination of the mechanisms underlying tsetse fly lactation. In general, our results show that the majority of genes have lower expression during lactation with the exception of those directly involved in milk production. The combination of transcriptomic and proteomic analyses reveals there are 12 major milk gland proteins, which comprise ∼47% of the transcriptome of lactating flies, along with multiple minor protein constituents of tsetse milk. We have provided an overview of the combined results of this study (Fig. 11). Furthermore, we discovered a novel, tsetse-specific protein family, MGP2–10, that is expressed highly during lactation. Interference with expression of these proteins reduces tsetse fly fecundity, suggesting that this family of MGP genes may provide a target for development of tsetse-specific abortifacients. This study has also revealed that many of the underlying functional aspects of tsetse fly lactation are analogous to those of other lactating organisms. This example of convergent evolution suggests that tsetse flies could be used as an invertebrate model system to investigate the complex molecular and physiological aspects associated with obligate lactation.

## Materials and Methods

### Flies

Colonies of *G. morsitans morsitans* were reared at Yale University and the Institute of Zoology at the Slovak Academy of Sciences (SAS). The other two species (*G. pallidipes* and *G. fuscipes*) were reared at SAS. Flies were maintained on blood meals provided through an artificial feeding system at 48 h intervals [104]. Two groups of females were used for transcriptome analysis: the first group carried a third instar larva (lactating) while the second group was examined 48 h post parturition (dry or non-lactating). Developing progeny were removed from each female to ensure transcript changes were representative of differences between the mothers. For sex specific transcript analysis, males and females were collected 16–18 d after adult emergence. Tissue samples were collected from pregnant females (16–18 d after adult emergence) carrying third instar larvae 24 h after blood feeding. Samples for temporal expression analyses were collected according to progeny development status based on previous studies [9], [47]. Flesh flies, *S. crassipalpis*, acquired from Ohio State University were reared according to standard procedures [105].

### RNA extraction and library preparation

Total RNA was extracted from individual flies or dissected tissues using Trizol reagent (Invitrogen, Carlsbad, CA, USA), following the recommended protocol. RNA was treated twice with the TURBO DNA Free kit (Ambion, Austin, TX, USA) to remove DNA, alcohol precipitated to remove residual salt, and further cleaned using the RNeasy kit (Qiagen, Maryland, USA). Total RNA (2–3 µg) was pooled from 10 flies extracted individually for each treatment. Sample quality and concentration was determined using a Bioanalyzer 2100 (Agilent, Palo Alto, CA, USA). Library construction was performed using standard protocols for Illumina mRNA-Seq sequencing by the W. M. Keck Foundation Microarray Resource at the Yale School of Medicine. Each single-end library was sequenced on one lane of the Genome Analyzer II platform (Illumina, San Diego, CA, USA).

### Transcriptome analysis of tsetse lactation samples

To determine Illumina read quality, FastQC analysis was performed on the transcriptomes generated from dry and lactating flies. Due to the prevalence of tsetse symbiont sequences in the reads, a specific quality control step was included to reduce bacterial sequence reads using the known whole genome sequence data from *Wigglesworthia* [106], *Wolbachia* (unpublished) and *Sodalis* [107] determined from the same host species *G. morsitans*. Following symbiont specific sequence removal, remaining sequences were trimmed in CLC Genomics (CLC Bio) to remove ambiguous nucleotides. Contig libraries were constructed using Abyss [16], [17] followed by a secondary assembly with Trinity [18]. Functional annotation was accomplished using the BLASTx algorithm through comparison with sequences included in the NCBI protein database [108] as well as the KOG [109] and GO databases [110]. Conserved protein domains were detected using rpsBLAST [111] searches against the CDD, Pfam and Smart databases [112]. Predicted protein translations were submitted to SignalP to identify potential secretion products by screening for secretion signal motifs [113]. Additionally, contigs were compared to several proteomes obtained from Flybase [114] (*D. melanogaster*) and Vectorbase [115] (*An. gambiae*). Each read from each library was compared by BLASTn to the assembled coding sequences (CDS) using a word size of 25, m8 output and low complexity filter turned off. CDS coverage and CDS number of read “hits” from each library were computed from the BLAST output file. A hit was only considered significant if it had 97% or better identity to its target and no more than one gap. The same read could be mapped up to three different CDS to the extent that their BLAST scores were identical. Expression levels were determined using CLC Genomics Workbench (CLC bio, Cambridge, MA). Reads were mapped to our *de novo* assembly with an algorithm allowing only two mismatches and a maximum of 10 hits per read. RPKM was used as a measure of gene expression [116]. The proportion of read counts for each contig in relation to the total read counts in each sample was determined in order to calculate P-value differences in proportions by a Z-test following Bonferroni correction [117]. Fold change was determined as the ratio of RPKM of lactating flies vs. RPKM of dry flies. In addition to the analysis of the complete Illumina libraries, a secondary analysis was conducted featuring Illuminia libraries filtered to eliminate milk-specific contigs to reduce bias by these highly abundant proteins [116], [117]. Data from this study are available in Sequence Read Archive (SRA075330).

### LC/MS/MS proteomic analysis of tsetse milk present in larval gut content

Pulled glass capillary tubes were used to collect milk samples by negative pressure from the guts of feeding third instar larvae, which were microscopically dissected from the uterus of pregnant females. Samples were stored in 1× protease inhibitor cocktail (Sigma-Aldrich). Proteins were precipitated with 10% trichloroacetic acid (Fisher Scientific) at 4°C overnight, collected by centrifugation (11,000×g, 30 minutes, 4°C) and washed two times with ice-cold acetone. Protein pellets were briefly dried and dissolved in 10 µl of protein pellet buffer (8M urea, 3M thiourea, and 1% dithiothreitol). Trypsin digestion was performed at 37°C for 12–16 h following dilution with distilled H_2_O to a final volume of 100 µl. Samples were stored at −80°C until analysis. Peptides were separated with a Waters nanoAcquity UPLC system (75 µm×150 mm BEH C18 eluted at 500 nl/min at 35°C) with Buffer A (100% water, 0.1% formic acid) and Buffer B (100% CH_2_CN, 0.075% formic acid). A linear gradient was established with 5% Buffer B, increasing to 50% Buffer B at 50 minutes and finally to 85% Buffer B at 51 minutes. MS/MS was acquired with an AB Sciex 5600 Triple Time-of-Flight mass spectrometer using 1 microscan followed by four MS/MS acquisitions. Neutral loss scans were obtained for 98.0, 49.0 and 32.7 amu. Seven separate 1 µl injections at an estimated 0.351 µg/µl concentration for a total of 2.457 µg on the column were used for analysis.

Mascot algorithm was used to analyze uninterrupted MS/MS spectra [118]. The Mascot Distiller program used MS/MS spectra to generate Mascot compatible files by combining sequential MS/MS scans from profile data that have the same precursor ion. Charge states of +2 and +3 were preferentially located with a signal-to-noise ratio of 1.2 or greater. A list of protein sequences was created and used in the BLASTx search against Trinity-assembled library from the pregnancy-specific analysis and positive matches were identified by tBLASTx against the NCBI and Swiss-Prot databases. Mascot scores were based on MOlecular Weight SEarch (MOWSE) relying on multiple matches of more than one peptide to the same predicted protein [119], [120]. The MOWSE based ions score is equal to (−10)*(Log_10_P), where P is the absolute probability that a match is random. Matches were considered significant when the probability of a random match fell below 5% (E value<0.05). Therefore, Mascot scores greater than 68 were above the significance threshold when searching the newly assembled library. Proteins were considered to be successfully identified when two or more peptides matched the same predicted protein and the Mascot score exceeded the significance threshold. The exponentially modified protein abundance index (empai) was employed to estimate levels of protein species based on the number of species detected compared to the number of possible peptides for specific protein [121], .

### Sequence analysis for novel milk protein family

Chromosomal organization of genes and full length mRNA sequences for *mgp2–10* were obtained by mapping Illumina high-throughput reads against *G. m. morsitans* genomic scaffolds in the CLC Genomics software package. Nucleotide and predicted protein sequences were aligned using PROMALS3D [123] and Clustal [124] and formatted with BioEdit [24]. Flesh fly, *Sarcophaga crassipalpis*, sequences were obtained from a previous EST project [20], [21]. Sequences of *mgp2–10* from other tsetse species (*G. pallidipes* and *G. fuscipes*) were obtained from female cDNA by RT-PCR followed by cloning into T-vector plasmid (Invitrogen) and sequenced at the DNA Analysis Facility at Yale University (New Haven, CT). Pairwise phylogenetic tree construction and bootstrap analysis (10000 replicates) were performed using the MEGA4/5 sequence analysis suite [25], [125]. dN/dS analyses were performed using the FEL (Fixed Effects Likelihood [28]) and PARRIS [27] algorithms available via DataMonkey [29], [30], which is a web-based implementation of the HyPhy phylogenetic analysis program [31]. Sequences were translated, aligned, reverse translated and the stop codons removed in accordance with the requirements for sequence input to DataMonkey. Under the FEL method, posterior probabilities cutoffs were set at 95, which is equivalent to a p-value of 0.05 for the site-specific detection of codons under positive or negative selection. Analysis of specific regions of the MGP2–10 coding regions was conducted using MEGA5 according to previous milk protein studies [32] and individual regions were based upon protein coding regions with high or low levels of amino acid homology.

### RNA isolation, RT-PCR and qPCR

For sex- and tissue-specific RT-PCR expression analyses, total RNA isolated from males and females and from dissected tissues was used as template for the Superscript III reverse transcriptase kit following the manufacturer's protocols (Invitrogen). Fat body and milk gland were analyzed as a combined samples since complete separation is nearly impossible due to the intricate association of these organs. PCR was performed with gene-specific primer pairs (Table S1) using the GoTaq DNA polymerase kit (Promega). The PCR amplification conditions were as follows: 95°C for 3 min, 35 cycles of 30 sec at 95°C, 52–56°C for 1 min, and 1 min at 70°C using a Bio-Rad DNA Engine Peltier Thermocycler (Hercules, CA).

For pregnancy-specific transcript abundance determination, qPCR analyses were performed using a CFX PCR detection system (Bio-Rad, Hercules). Data were analyzed with CFX manager software version 3.1 (Bio-Rad). Primer sequences used were the same as used in RT-PCR analyses (Table S1). Comparative Ct values for genes of interest were standardized by Ct values for the control gene (*tubulin*) relative to the average value for the control treatment or newly emerged flies, yielding the delta Ct value. All experiments were analyzed in triplicate and subject to ANOVA followed by Bonferroni correction and Dunnett's test.

### RNA interference of MGP family

Short interfering RNAs (siRNA) consisting of two Duplex sequences (Table S1) were designed using Integrated DNA Technologies online software (IDT). Control siRNAs were designed against Green Fluorescent Protein (GFP; Table S1). Each oligo, designed to target a single *mgp* gene, was also compared to the reference RNA library/*G. morsitans* genome [14]) and the Trinity contigs library from this study to verify target specificity. The oligos for each strand of the siRNA were combined, and the concentration was determined spectrophotometrically followed by adjustment to 800–850 ng/µl per siRNA. Each female fly was injected with 2 µl siRNA solution into the thorax 8–10 d after adult emergence. Five days post-injection, gene expression levels were determined by qPCR and normalized to *tubulin* transcripts. For combined knockdown studies, siMGP constructs were mixed to yield a sample concentration of at least 600 ng/µl for each siRNA targeting a specific MGP transcript. Fecundity following MGP knockdown was assessed as previously described [9]. Finally, milk protein content was determined by Bradford assay (Bio-Rad) after extraction from the larval gut contents as described above.

### Lipid emulsification assays

Emulsification assays were based on milk turbidity measurements. For this assay, milk was acquired from the guts of actively feeding larvae as before and diluted 10× prior to the assay. Samples were vortexed for 1 min at 10,000 rpm, and absorbance of the diluted emulsion was measured at 500 nm. Changes in absorbance were measured hourly for 10 h. Results were analyzed based on the slope of a regression, where ln (ABS_t_/ABS_0_) is plotted versus time based on the exponential model (ABS_t_ = ABS_0_ e^−kt^). For this model, ABS_t_ denotes absorbance at any time t, ABS_0_ is the initial absorbance, and k is the rate of absorbance decline in %/h.

### Protein structure prediction

To generate structural models for MGP2–10, four web-based *de novo* protein modeling programs were consulted. QUARK is a recently developed *ab initio* assembly program that will first break proteins into small sequences, following which full-length sequence models are assembled using Monte Carlo simulations [126]. The I-TASSER program first develops a three-dimensional model and subsequently predicts function based on structural similarity with functionally defined proteins [127]. Phyre2 is a widely used protein homology/analogy recognition engine that can rapidly predict the structure of 250 residue proteins [128]. Finally, SPARKS-X is a program that performs well in comparison to other programs [129]. Each program was run under the default configuration and the resultant predicted protein structures were visualized using Discovery Studio 3.1 (Accelrys).

## Supporting Information

Figure S1 Length of contigs over 200 bp generated by the combination of Abyss [16], [17] and Trinity [18] de novo assembly program.
(TIF)

Figure S2 Distribution of reads per contig in RNA-seq libraries with at least 2 mapped reads and 25 total reads between the two sample sets. Lactating (A) and dry (B) contigs are displayed in descending amount of number of reads per contig.
(TIF)

Figure S3 Time course of *28S* expression during the first two tsetse gonotrophic cycles. Transcript levels were determined by qPCR with a CFX PCR detection system (Bio-Rad, Hercules) and data were analyzed with CFX manager software version 3.1 (Bio-Rad). Data represent the mean ± SE of three replicates and was normalized to *tubulin*.
(TIF)

Figure S4 Summary of specific genes that are differentially expressed in lactating compared to dry flies. (A) Antioxidant enzyme genes (*superoxide dismutase*, *SOD*). (B) Heat shock protein genes. (C) Lipid metabolism genes (*Forkhead Box Sub Group O*, *FOXO*; *Histone Deacetylase 4*, *hdac4*; *monoacylglycerol O-acyltransferase*, *mogat*). (D) Yolk protein genes. *, indicates significantly different between lactating and dry flies based on Kal's test followed by Bonferroni correction.
(TIF)

Figure S5 Amino acid analysis of MGP2–10. Multiple alignment of full length amino acid sequences of *Glossina morsitans* milk gland protein 2–10 (MGP2–10) and *Sarcophaga crassipalpis* milk gland protein-like protein (Sc-MGP-like protein). Multiple alignment was performed with ClustalX and optimized in BioEdit. Blue indicates at least 75% similarity between sequences and gray indicates 75% similarity between the classes of amino acids. Blue line above alignment indicates region of high sequence similarity.
(TIF)

Figure S6 Amino acid phylogeny of partial overlapping region of *Glossina morsitans morsitans*, *G. pallidipes* and *G. fuscipes* milk gland proteins and *Sarcophaga crassipalpis* milk gland protein-like protein amino acid sequences. (A) Initial sequence alignment was completed using PROMALS3D server (PROfile Multiple Alignment with predicted Local Structures and 3D constraints) [123] and ClustalX [23], [124] and formatted with BioEdit [24]. (B) Evolutionary analyses were conducted in MEGA4 [25], [26], [125].
(TIF)

Figure S7 Predicted protein structure of *Glossina morsitans morsitans*. Structures predicted by Quark (126), I-TASSER [127], SPARK-X [129] and Phyre [128].
(TIF)

Figure S8 Transcript levels of milk gland protein and milk gland protein-like genes in *G. fuscipes*, *G. pallidipes* and the flesh fly, *Sarcophaga crassipalpis* in relation to total RNA content. (A) Transcript levels in tsetse mother with the indicated progeny developing in the ovary or uterus (B) Transcript levels in the flesh fly. Transcript levels were determined by qPCR. Data represent the mean ± SE of three replicates and was normalized to *nadh subunit 2* (*G. fuscipes*), *28S* (*G. pallidipes*) and *Rp49* (*S. crassipalpis*). *, denotes significant difference from control following ANOVA with Tukey's test at P<0.01.
(TIF)

Table S1 Quantitative PCR primers and siRNA used for injection.
(XLSX)

Table S2 Complete results for RNA-seq analysis comparing lactating and dry tsetse flies, *Glossina morsitans*.
(XLSX)

Table S3 Results of RNA-seq analysis with statistical differences between lactating and dry determined by a Kal's test with Bonferroni correction, P<0.05.
(XLSX)

Table S4 Trypsin genes decreased in dry flies in comparison to lactating flies.
(XLSX)

Table S5 Results for RNA-seq data comparing lactating and dry flies following removal of reads for milk-specific contigs.
(XLSX)

Table S6 Results of RNA-seq data with statistical differences between lactating and dry flies determined by a Kal's test with Bonferroni correction, P<0.05 following removal of reads for milk-specific contigs.
(XLSX)

Table S7 Complete results for proteomic analysis of tsetse milk secretion.
(XLSX)

Table S8 Amino acid composition of milk gland proteins 2–10 as percent amino acids per protein. Total represents the average percent across MGP2–10. Highlighted are the essential amino acids.
(XLSX)

Text S1 Validation of RNA-seq data with qPCR. Correlation of log_2_ ratios from RNA-seq and qPCR values for seventeen genes. The Pearson's correlation coefficient (0.934) and goodness of fit (R^2^ = 0.872) were high, indicating a high degree of correlation between RNA-seq and qPCR fold changes between dry and lactating flies.
(DOCX)
