# Supplementary figures and images for: A Novel Highly Divergent Protein Family Identified from a Viviparous Insect by RNA-seq Analysis: A Potential Target for Tsetse Fly-Specific Abortifacients
Source: PLoS Genet. 2014 Apr 24;10(4):e1003874. doi: 10.1371/journal.pgen.1003874 (PMC3998918; doi:10.1371/journal.pgen.1003874)

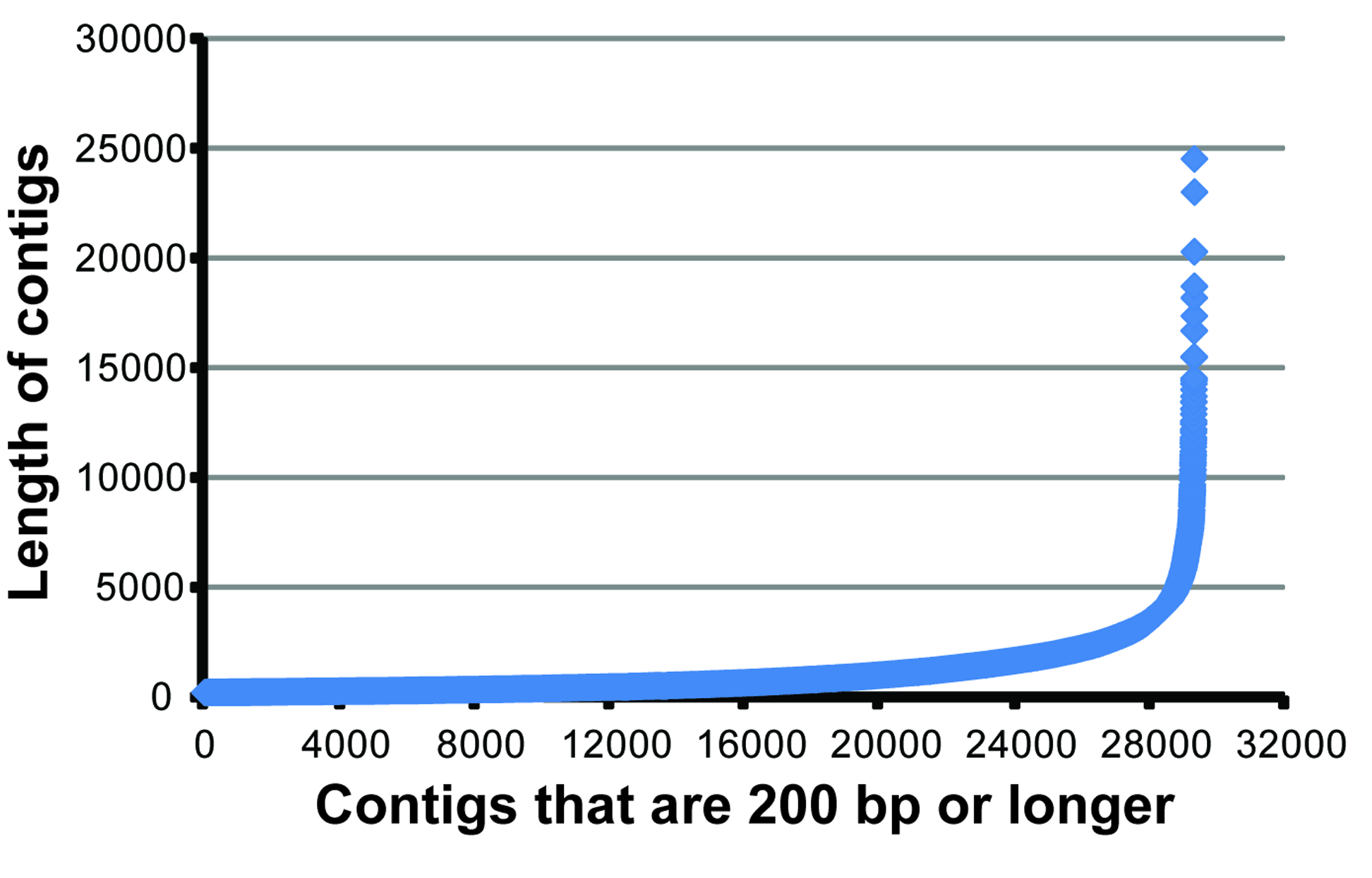

Supplement: Figure S1 — Length of contigs over 200 bp generated by the combination of Abyss [16], [17] and Trinity [18] de novo assembly program. (TIF) [file pgen.1003874.s001.tif]

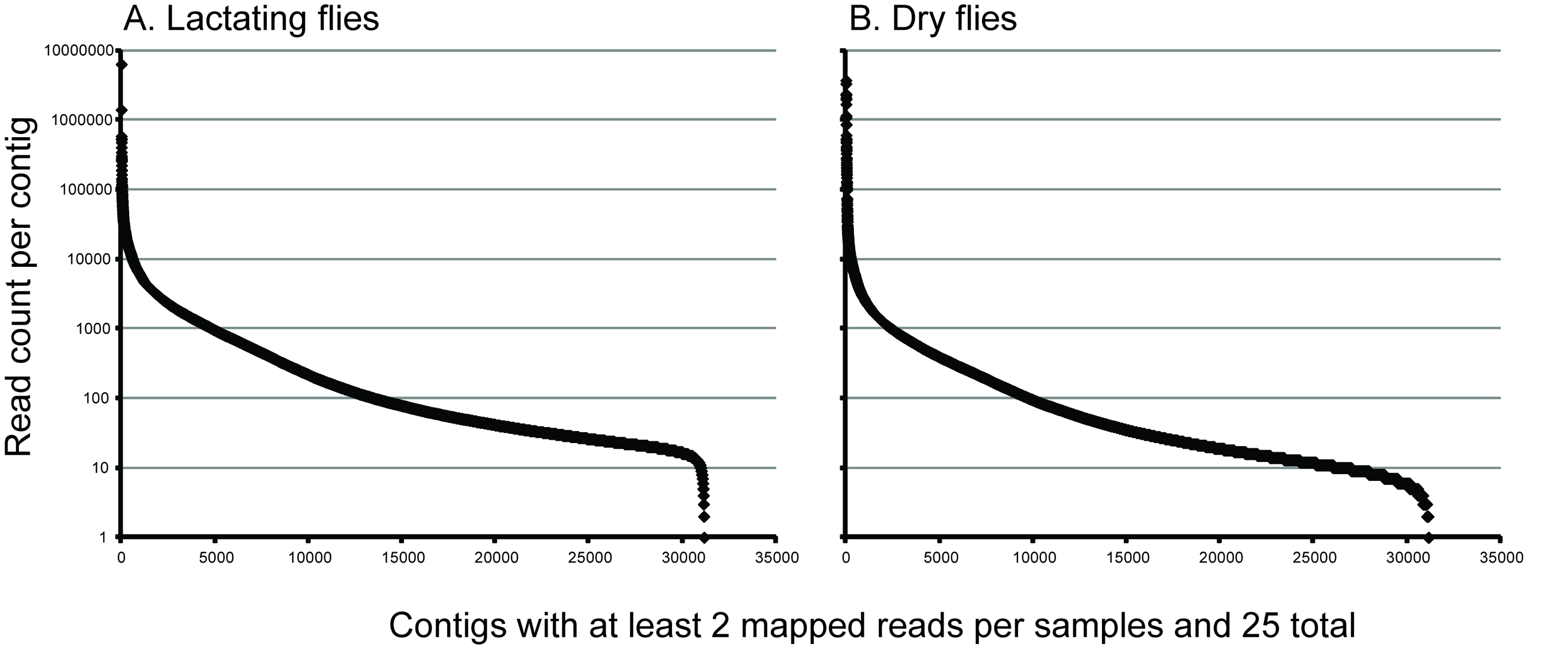

Supplement: Figure S2 — Distribution of reads per contig in RNA-seq libraries with at least 2 mapped reads and 25 total reads between the two sample sets. Lactating (A) and dry (B) contigs are displayed in descending amount of number of reads per contig. (TIF) [file pgen.1003874.s002.tif]

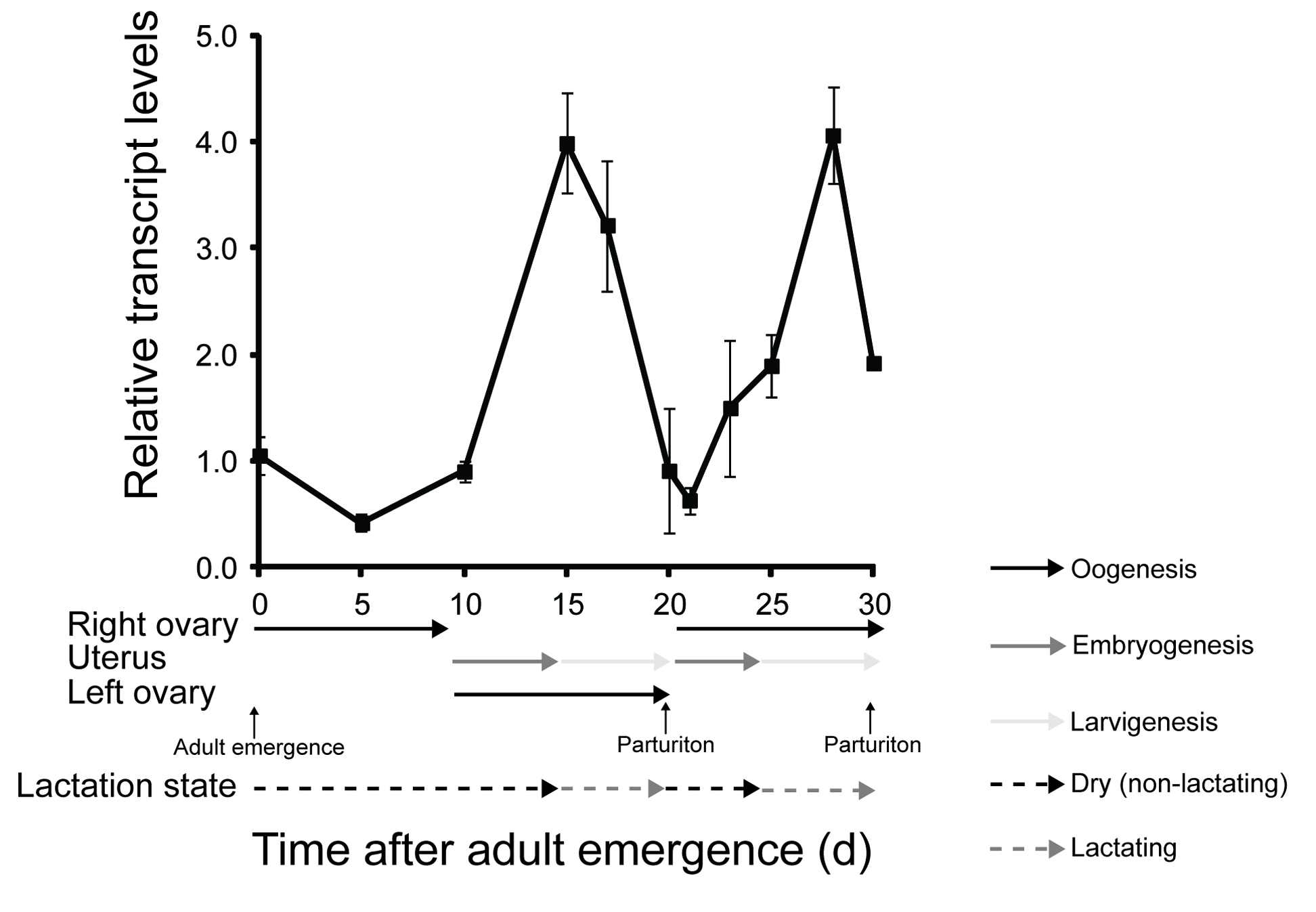

Supplement: Figure S3 — Time course of 28S expression during the first two tsetse gonotrophic cycles. Transcript levels were determined by qPCR with a CFX PCR detection system (Bio-Rad, Hercules) and data were analyzed with CFX manager software version 3.1 (Bio-Rad). Data represent the mean ± SE of three replicates and was normalized to tubulin. (TIF) [file pgen.1003874.s003.tif]

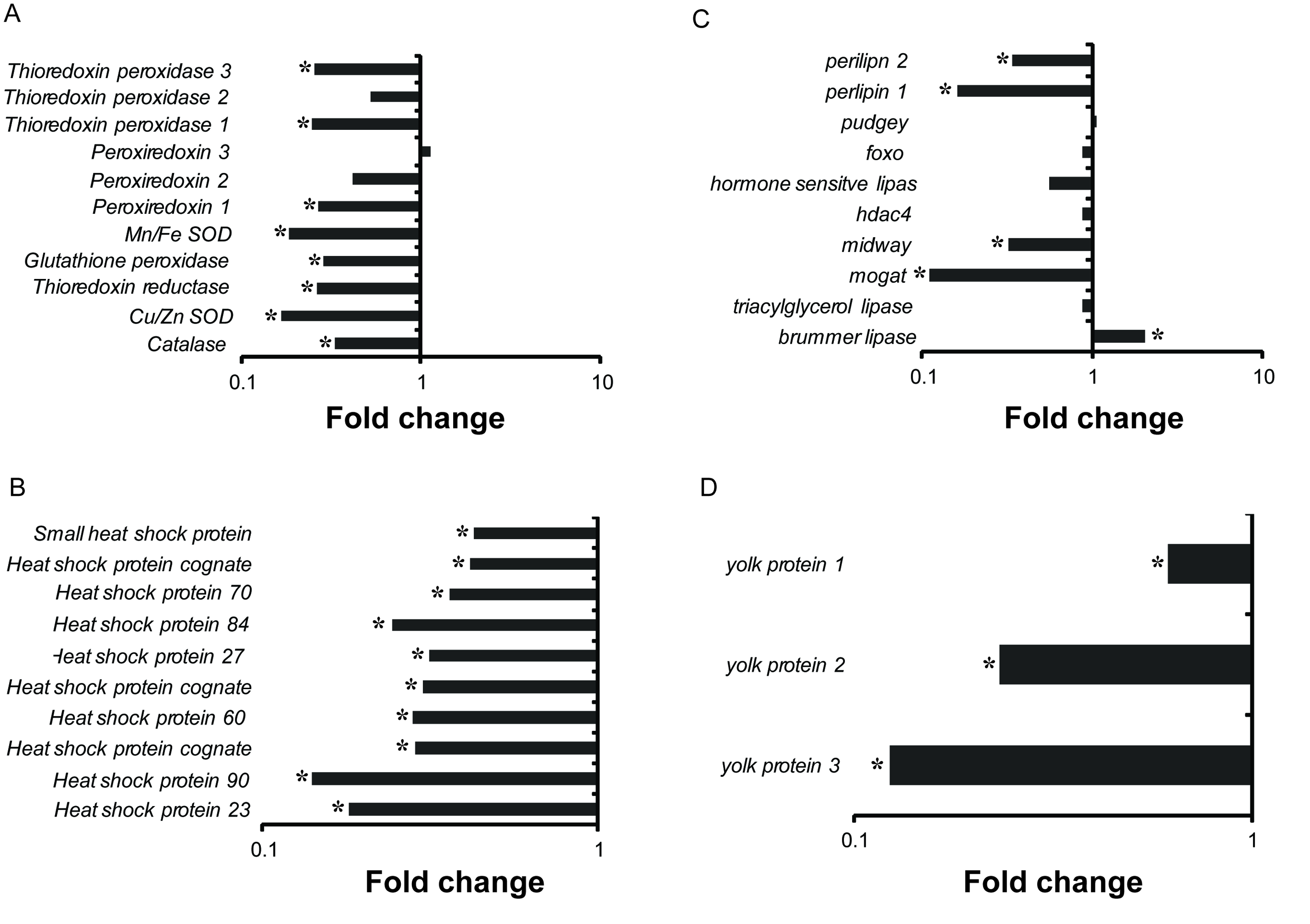

Supplement: Figure S4 — Summary of specific genes that are differentially expressed in lactating compared to dry flies. (A) Antioxidant enzyme genes (superoxide dismutase, SOD). (B) Heat shock protein genes. (C) Lipid metabolism genes (Forkhead Box Sub Group O, FOXO; Histone Deacetylase 4, hdac4; monoacylglycerol O-acyltransferase, mogat). (D) Yolk protein genes. *, indicates significantly different between lactating and dry flies based on Kal's test followed by Bonferroni correction. (TIF) [file pgen.1003874.s004.tif]

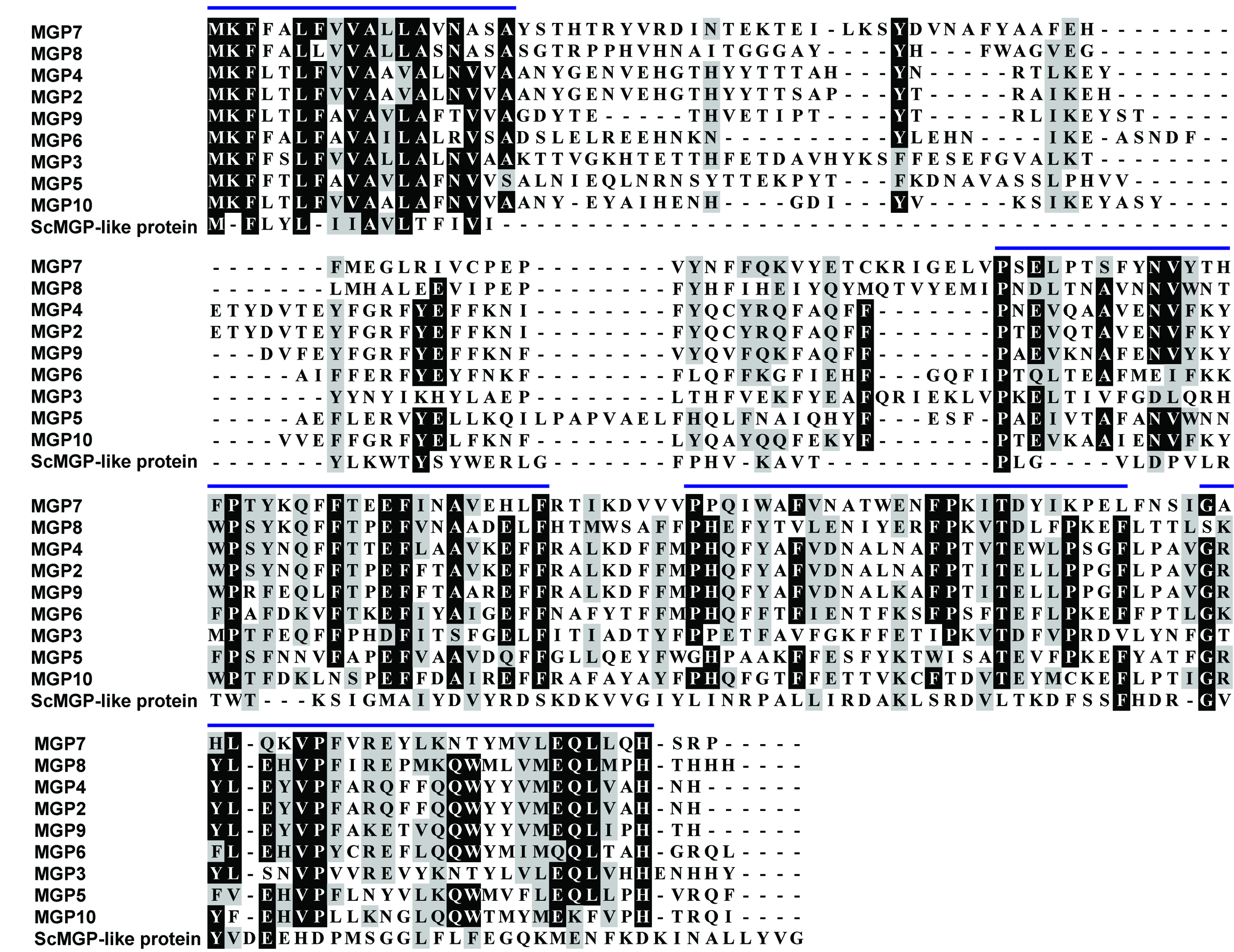

Supplement: Figure S5 — Amino acid analysis of MGP2–10. Multiple alignment of full length amino acid sequences of Glossina morsitans milk gland protein 2–10 (MGP2–10) and Sarcophaga crassipalpis milk gland protein-like protein (Sc-MGP-like protein). Multiple alignment was performed with ClustalX and optimized in BioEdit. Blue indicates at least 75% similarity between sequences and gray indicates 75% similarity between the classes of amino acids. Blue line above alignment indicates region of high sequence similarity. (TIF) [file pgen.1003874.s005.tif]

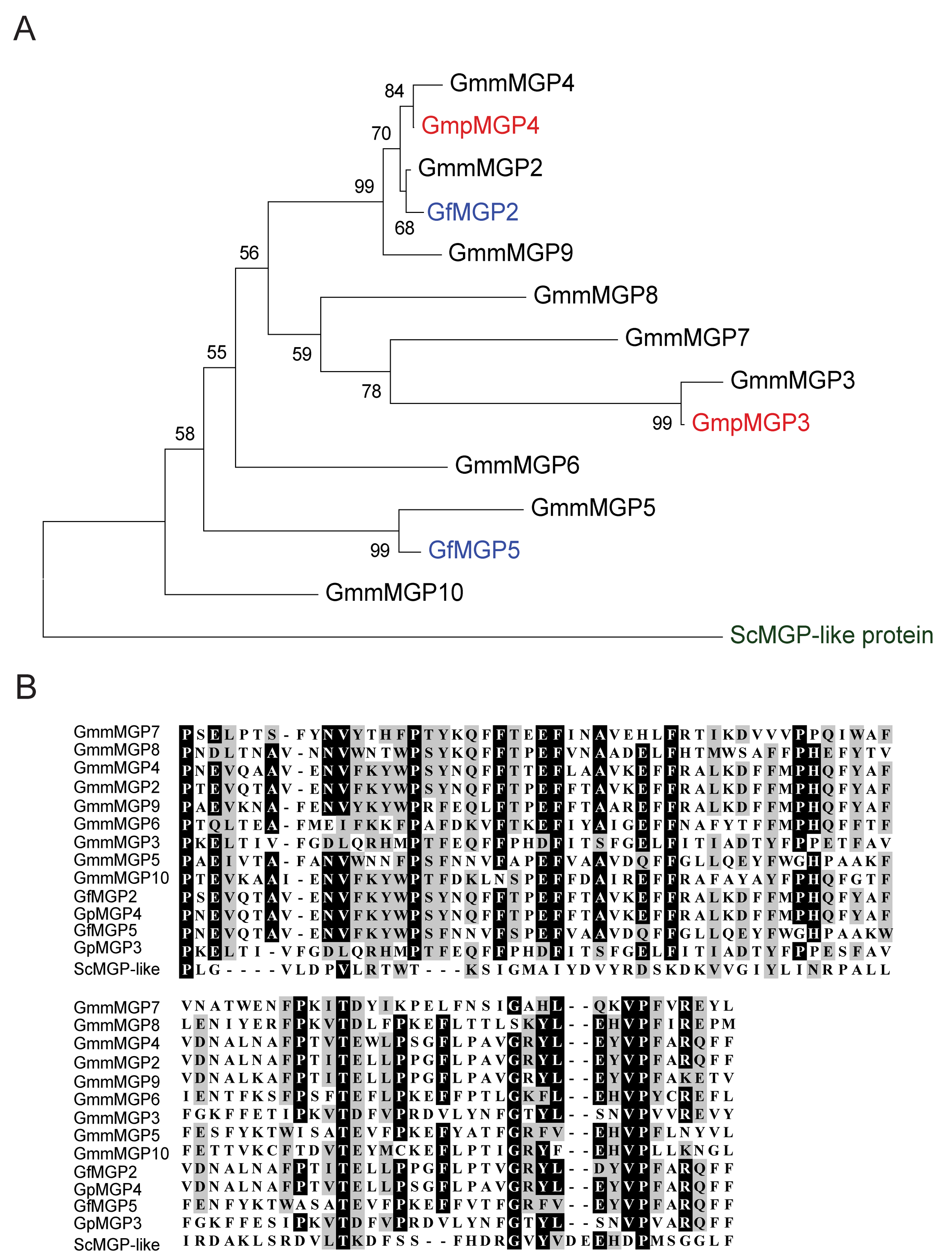

Supplement: Figure S6 — Amino acid phylogeny of partial overlapping region of Glossina morsitans morsitans, G. pallidipes and G. fuscipes milk gland proteins and Sarcophaga crassipalpis milk gland protein-like protein amino acid sequences. (A) Initial sequence alignment was completed using PROMALS3D server (PROfile Multiple Alignment with predicted Local Structures and 3D constraints) [123] and ClustalX [23], [124] and formatted with BioEdit [24]. (B) Evolutionary analyses were conducted in MEGA4 [25], [26], [125]. (TIF) [file pgen.1003874.s006.tif]

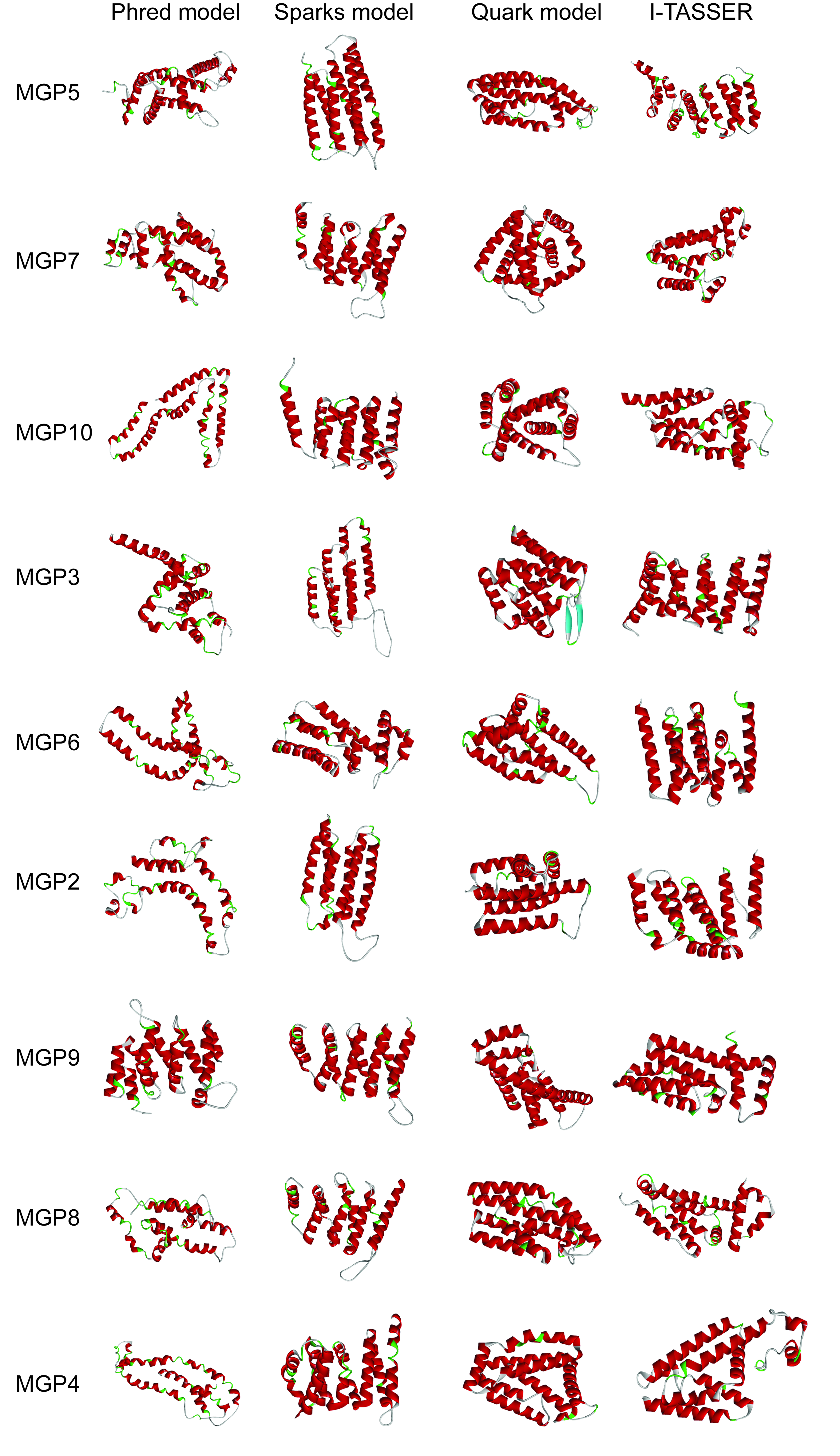

Supplement: Figure S7 — Predicted protein structure of Glossina morsitans morsitans. Structures predicted by Quark (126), I-TASSER [127], SPARK-X [129] and Phyre [128]. (TIF) [file pgen.1003874.s007.tif]

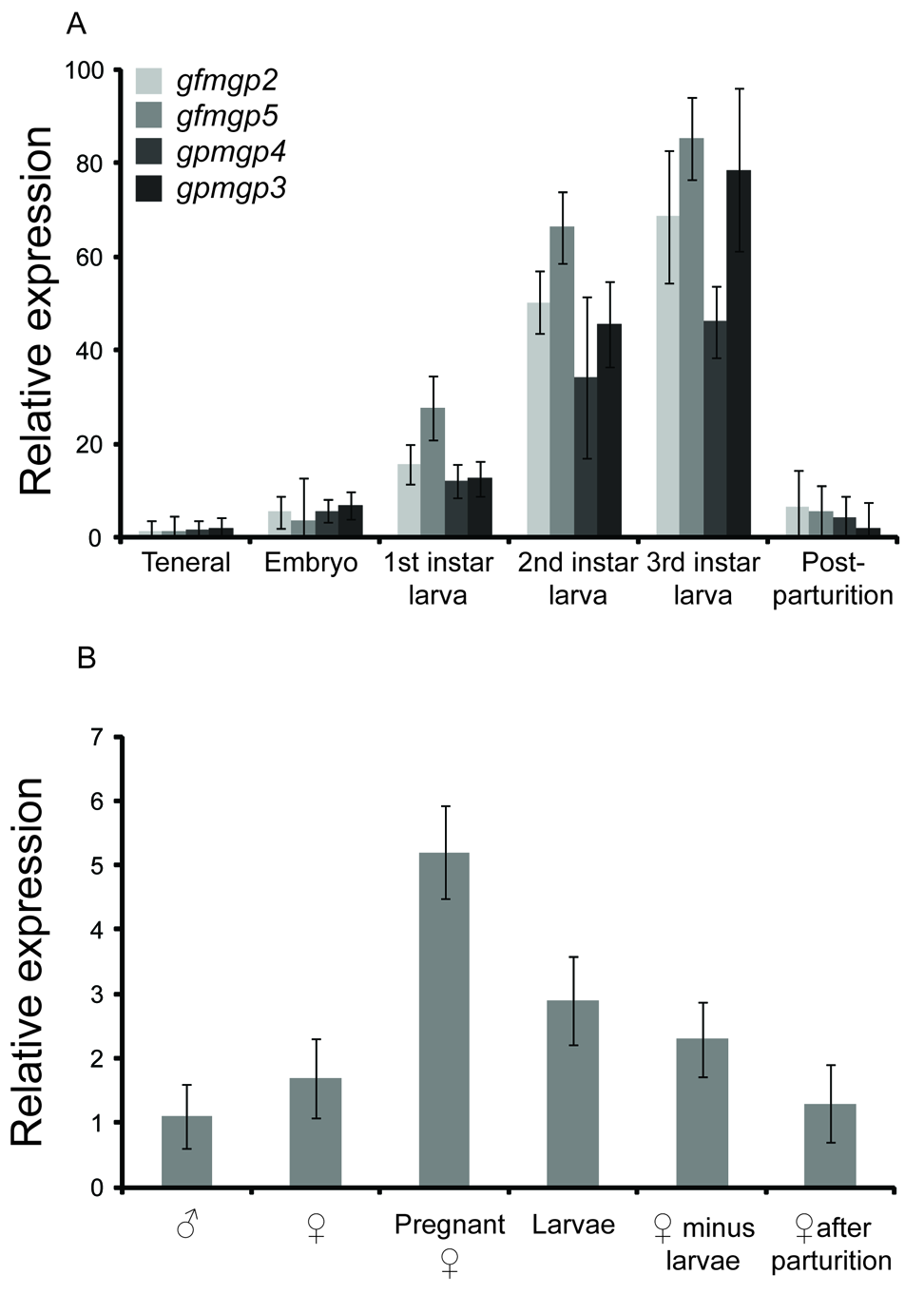

Supplement: Figure S8 — Transcript levels of milk gland protein and milk gland protein-like genes in G. fuscipes, G. pallidipes and the flesh fly, Sarcophaga crassipalpis in relation to total RNA content. (A) Transcript levels in tsetse mother with the indicated progeny developing in the ovary or uterus (B) Transcript levels in the flesh fly. Transcript levels were determined by qPCR. Data represent the mean ± SE of three replicates and was normalized to nadh subunit 2 (G. fuscipes), 28S (G. pallidipes) and Rp49 (S. crassipalpis). *, denotes significant difference from control following ANOVA with Tukey's test at P<0.01. (TIF) [file pgen.1003874.s008.tif]
