# Supplementary material for: A Novel Highly Divergent Protein Family Identified from a Viviparous Insect by RNA-seq Analysis: A Potential Target for Tsetse Fly-Specific Abortifacients
Source: PLoS Genet. 2014 Apr 24;10(4):e1003874. doi: 10.1371/journal.pgen.1003874 (PMC3998918; doi:10.1371/journal.pgen.1003874)
Supplement: Text S1 — Validation of RNA-seq data with qPCR. Correlation of log2 ratios from RNA-seq and qPCR values for seventeen genes. The Pearson's correlation coefficient (0.934) and goodness of fit (R2 = 0.872) were high, indicating a high degree of correlation between RNA-seq and qPCR fold changes between dry and lactating flies. (DOCX) [file pgen.1003874.s017.docx]

**Text S1**

**Validation of RNA-seq results with qPCR**. The RNA-seq expression values (log_2_ ratios) for seventen genes plotted against qPCR values (log_2_ ratios). The Pearson correlation coefficient (R=0.976) and goodness of fit (R^2^= 0.954) were high indicating a high degree of corelation. This indictes the qPCR validates the RNA-seq data.

**
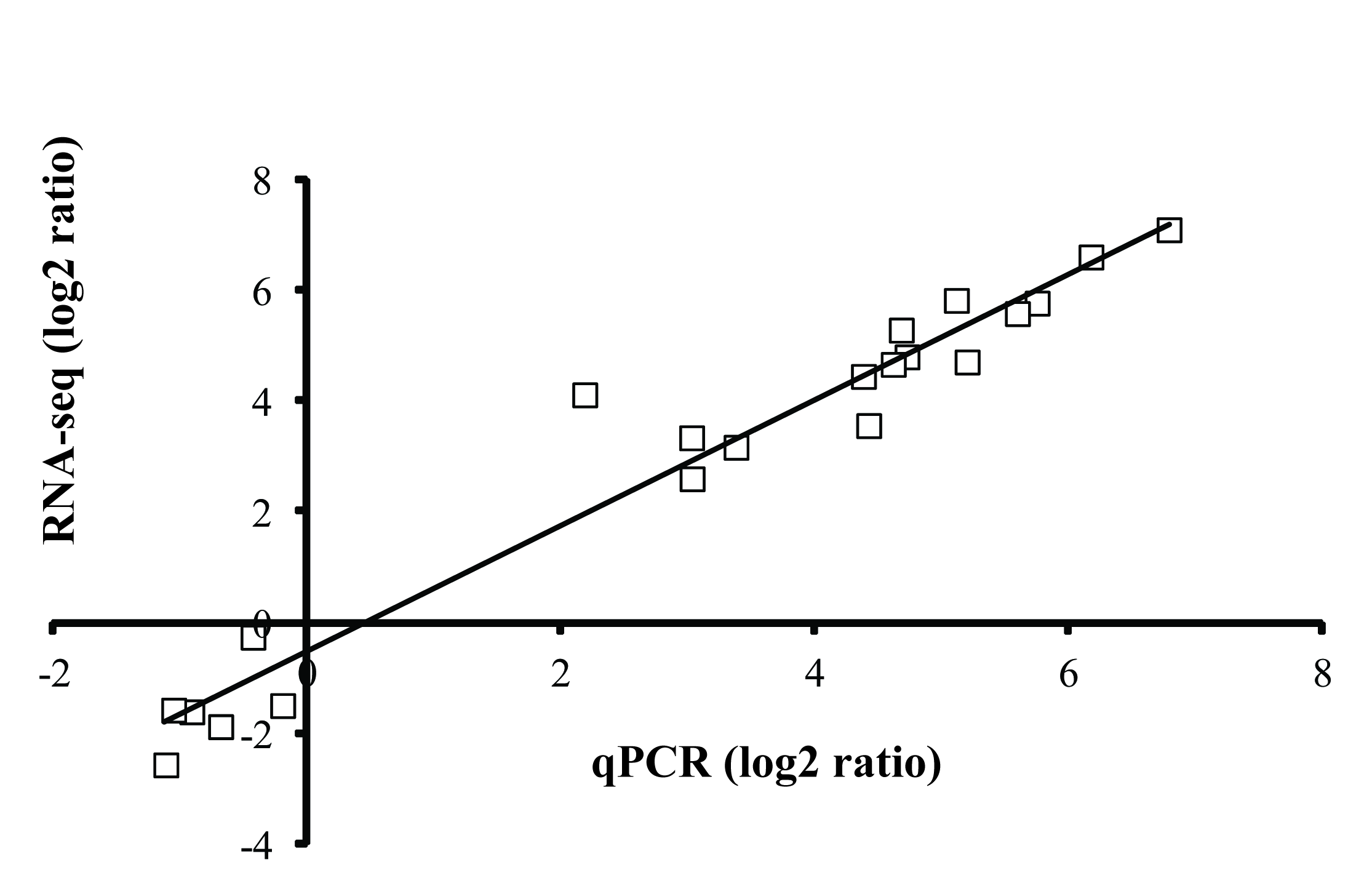
**
